# Increased Brown Adipose Tissue Thermogenesis in Phenylketonuria

**DOI:** 10.1002/mco2.70820

**Published:** 2026-06-13

**Authors:** Noemí López‐Rey, Alba Cabaleiro, María P. Pata, Marion Peyrou, Ánxela Estévez‐Salguero, Paola Fernández‐Sanmartín, Donald A. Morgan, Vitor Ferreira, Paula Sánchez‐Pintos, Cintia Folgueira, Carlos Diéguez, Adela Urisarri, Ismael González‐García, Luisa M. Seoane, Kamal Rahmouni, Francesc Villarroya, María L. Couce, Rubén Nogueiras, Miguel López

**Affiliations:** ^1^ Department of Physiology, CIMUS University of Santiago de Compostela Santiago de Compostela Spain; ^2^ Department of Pediatrics, Neonatology Service, University Clinical Hospital of Santiago de Compostela Instituto de Investigación Sanitaria de Santiago de Compostela (IDIS) and Spanish Network in Maternal Neonatal, Child and Developmental Health Research (RICORS‑SAMID) Santiago de Compostela Spain; ^3^ Research Unit Hospital Lucus Augusti Lugo Spain; ^4^ CIBER Fisiopatología de la Obesidad y Nutrición (CIBEROBN) Santiago de Compostela Spain; ^5^ Biostatech Advice Training and Innovation in Biostatistics S.L. Ames Spain; ^6^ Departament de Bioquímica i Biomedicina Molecular Institut de Biomedicina de la Universitat de Barcelona (IBUB) and Institut de Recerca Hospital Sant Joan de Déu Barcelona Spain; ^7^ Department of Neuroscience and Pharmacology University of Iowa Iowa City Iowa USA; ^8^ Neuroendocrine Regulation of Metabolism Group Instituto de Investigación Sanitaria de Santiago de Compostela (IDIS) Santiago de Compostela Spain; ^9^ Grupo Fisiopatología Endocrina Área de Endocrinología Instituto de Investigación Sanitaria de Santiago de Compostela (IDIS) Santiago de Compostela Spain; ^10^ Veterans Affairs Health Care System Iowa City Iowa USA; ^11^ CIBER Enfermedades Raras (CIBERER) Travesía Choupana Santiago de Compostela Spain

**Keywords:** AMP‑activated protein kinase (AMPK), brown adipose tissue (BAT), fibroblast growth factor 21 (FGF21), hypothalamus, phenylketonuria (PKU), thyroid hormones (THs)

## Abstract

Phenylketonuria (PKU), the most common autosomal‑recessive disorder of amino acid metabolism, is characterized by neurological impairment and systemic metabolic alterations caused by chronically elevated phenylalanine (Phe) levels. PKU patients have long been reported to display reduced metabolic rate and impaired thermoregulation, yet the role of brown adipose tissue (BAT) in this condition remains unknown. Here, noninvasive infrared thermography was used to assess BAT activity in a cohort primarily comprising children and adolescents including controls, mild hyperphenylalaninemia (MHPA), and PKU patients, while circulating metabolic and hormonal parameters were analyzed for associations with BAT temperature. Despite overall normothermia, individuals with PKU exhibited higher BAT temperature than both control and MHPA patients, which correlated with circulating fibroblast growth factor 21 (FGF21) and thyroid hormones. To gain mechanistic insight, rats and mice were centrally treated with FGF21, reproducing the BAT thermogenic phenotype along with decreased hypothalamic AMP‑activated protein kinase (AMPK) activity and increased sympathetic drive to BAT. Consistently, analysis of public single‑cell RNA‑sequencing data revealed convergent expression of AMPK, thyroid hormone receptor, and FGF21 receptor signaling in specific hypothalamic neuronal populations. These findings reveal enhanced BAT thermogenesis in PKU and demonstrate that Phe‐induced FGF21 disrupts energy homeostasis via hypothalamic AMPK inhibition.

## Graphical abstract

In phenylketonuria (PKU), elevated phenylalanine (Phe) increases hepatic fibroblast growth factor 21 (FGF21) and thyroid hormones, enhancing brown adipose tissue (BAT) thermogenesis in patients. Central FGF21 reproduces this phenotype in rodents via reduced hypothalamic AMP‐activated protein kinase (AMPK) activity in the ventromedial nucleus of the hypothalamus (VMH) and increased sympathetic nervous system (SNS) drive to BAT. Image created with BioRender.

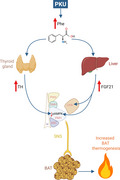

## 1 Introduction

Phenylketonuria (PKU; Online Mendelian Inheritance in Man (OMIM): 261600) is the most common autosomal recessive Mendelian disease of amino acid metabolism. It has been estimated that 0.45 million individuals have PKU worldwide [1, 2, 3, 4]. PKU is caused in 98%–99% of cases by deficiency of the hepatic enzyme L‐phenylalanine‐4‐hydroxylase (PAH; EC 1.14.16.1), which converts phenylalanine (Phe) to tyrosine (Tyr). The remaining 1%–2% of the cases are due to deficiencies in the cofactor tetrahydrobiopterin (BH_4_), which acts as a coenzyme [1, 2, 3, 4].

The clinical picture of PKU is highly varied, depending on the degree of residual PAH activity, as well as brain and blood Phe concentrations in patients. Lower residual enzyme activity usually results in higher blood Phe concentrations and a more severe clinical outcome if untreated [1, 2, 3, 4]. According to European guidelines (Section 4.6), PAH deficiency is considered as mild hyperphenylalaninemia (MHPA) with Phe levels of 120–< 360 µmol/L (1.98–< 5.94 mg/dL), which does not require treatment, and PKU, with levels ≥ 360 µmol/L (≥ 5.94 mg/dL; both cofactor‐responsive and ‐unresponsive), requiring treatment [5]. Untreated PKU generally results in overall developmental delay, microcephalia, severe permanent intellectual disability, as well as impaired growth, hypopigmentation, motor deficits, ataxia, and seizures [1, 2, 3, 4]. Early diagnosis and treatment with a low‐Phe diet enables improvement of life for the majority of PKU patients [1, 2, 3, 4]. Pharmacological treatment with sapropterin dihydrochloride, a synthetic formulation of BH_4_, and enzyme substitution therapy with Phe ammonia lyase (PAL) also provides alternative treatment options for some PKU patients [1, 2, 3, 4].

The impact of PKU on the regulation of energy balance is unclear. Obesity prevalence in people with PKU is comparable to that of the general population [6, 7]. Several studies have also investigated the basal and stimulated metabolic rate in PKU, but the findings have been inconsistent ranging from no detectable effect to reports of reduced metabolic rate in PKU patients [6, 8]. It is noteworthy that thermoregulation is impaired in PKU, with patients sweating minimally in hot environments and, of note, failing to properly defend body temperature after cold exposure [9]. This raises the question of how adaptive thermogenesis is regulated in PKU. Although vasodilation, vasoconstriction, and shivering occur normally in individuals with PKU [9], brown adipose tissue (BAT) thermogenesis has not yet been thoroughly investigated in this condition either in adult of pediatric populations [10, 11, 12]. This is surprising for several reasons: (i) first, *N*‐acyl amino acids, such as Phe, are potent mitochondrial uncouplers in human adipocytes, providing a molecular basis for an increased thermogenic rate; (ii) second, PKU patients show a trend to elevated circulating levels of thyroid hormones (THs): free triiodothyronine (fT3) and free thyroxine (fT4) [13, 14], both well‐known thermogenic agents [15]; and (iii) that BAT prevalence and activity increase during puberty, with notable sex differences and strong regulation by hormonal changes associated with sexual maturation [16, 17, 18, 19, 20, 21, 22, 23].

Thus, given the metabolic and thermoregulatory alterations associated with PKU, the aim of this study has been to investigate BAT thermogenesis in patients with PKU and with MHPA. To gain mechanistic molecular insight, we also analyzed the effect of Phe on hepatic and adipose human cells in vitro and central fibroblast growth factor 21 (FGF21) administration in rodents to probe hypothalamic control of BAT.

## 2 Results

### 2.1 BAT Temperature Is Increased in PKU Patients Despite Normothermia

**FIGURE 1:**
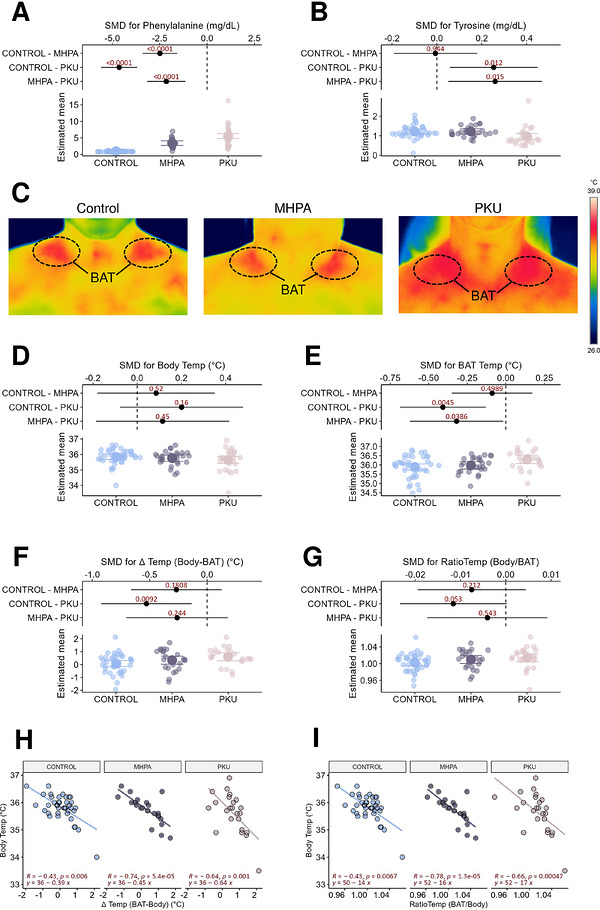
Effect of PKU on BAT temperature. (A) Phenylalanine (Phe) and (B) tyrosine (Tyr) circulating levels, (C) representative thermal images, (D) body temperature, (E) brown adipose tissue (BAT) temperature, (F) gradient BAT temperature minus body temperature, (G) ratio BAT temperature/body temperature and correlations between (H) body temperature and temperature gradient and (I) body temperature and temperature ratio in control, mild hyperphenylalaninemia (MHPA) and phenylketonuria (PKU) patients. Differences between groups was determined by contrast of marginal means estimated by linear regression. Association analysis was performed by Pearson's test. Alpha level was set at 0.05. All statistical tests were two‐sided. SMD, standardized mean difference.

We aimed to determine whether PKU was associated with altered BAT thermogenesis. A cohort of 86 White patients (median age, 11.0 years [Q1: 6.96, Q3: 14.75]; range, 2–23 years) was used for this study (Table 1). Analysis of circulating Phe confirmed the genetic diagnosis, demonstrating that MHPA and PKU groups showed higher concentrations of Phe than controls (Figure 1A and Table S1). On the other hand, circulating Tyr levels were decreased in the PKU group when compared with both controls and MHPA patients, among whom no significant differences were observed (Figure 1B and Table S1). Despite decreased Tyr concentrations in the PKU group, the circulating levels of catecholamines were normal in these patients (Figure S1A–C and Table S1). Moreover, catecholamine‑to‑Tyr ratios (dopamine/Tyr, noradrenaline/Tyr, adrenaline/Tyr) were calculated to account for precursor availability. Both dopamine/Tyr and noradrenaline/Tyr ratios were elevated in PKU patients compared with controls and MHPA, whereas the adrenaline/Tyr ratio was unchanged, (Table S1), suggesting altered peripheral catecholamine‐to‐tyrosine relationships in PKU.. Circulating alanine and methionine were also measured, as routinely monitored amino acids in PKU. Methionine levels were significantly lower in PKU than in both controls and MHPA, whereas alanine did not differ significantly between groups (Table S1).

**TABLE 1:** Anthropometric and nutritional parameters of participants.

	Control (*N* = 39)	MHPA (*N* = 23)	PKU (*N* = 24)	Test	Statistical	*p*
**Sex**						
Men	18 (46.15%)	6 (26.09%)	12 (50.00%)	Chi‐sq	*χ* ^2^ (2) = 3.300	0.192
Women	21 (53.85%)	17 (73.91%)	12 (50.00%)			
**Age (years)**						
Mean (SD)	11.45 (5.06)	10.91 (4.02)	10.38 (5.06)	ANOVA	F (2, 83) = 0.370	0.692
Median (Q1, Q3)	11.67 (6.96, 15.25)	11.00 (8.67, 14.50)	9.08 (6.83, 14.69)			
Range	3.17 23.00	2.67 15.92	2.00 20.00			
**Height (cm)**						
Mean (SD)	145.97 (25.51)	146.00 (22.61)	135.42 (25.23)			
Median (Q1, Q3)	153.50 (122.50, 163.50)	154.00 (138.35, 160.30)	132.50 (118.88, 160.00)	Kruskal–Wallis	*χ* ^2^ (2) = 2.565	0.277
Range	100.00 185.00	91.50 176.00	87.50 177.00			
**Body weight (kg)**						
Mean (SD)	42.76 (21.59)	45.37 (19.49)	38.34 (19.93)			
Median (Q1, Q3)	42.30 (23.80, 51.00)	47.80 (30.40, 54.20)	29.20 (21.45, 59.15)	Kruskal–Wallis	*χ* ^2^ (2) = 1.005	0.605
Range	15.20 109.20	15.70 96.10	12.20 72.20			
**BMI (kg/m^2^)**						
Mean (SD)	18.76 (3.92)	20.24 (4.67)	18.30 (4.13)			
Median (Q1, Q3)	18.20 (15.80, 19.85)	19.00 (17.98, 23.10)	16.45 (14.97, 22.38)	Kruskal–Wallis	*χ* ^2^ (2) = 2.912	0.233
Range	14.30 32.30	13.20 31.26	13.30 28.60			
**MME (kg)**						
Mean (SD)	17.69 (10.27)	20.20 (14.63)	14.92 (9.65) (*N* = 23)			
Median (Q1, Q3)	17.00 (9.05, 22.70)	19.50 (13.25, 21.70)	12.30 (7.70, 24.00)	Kruskal–Wallis	*χ* ^2^ (2) = 1.396	0.498
Range	1.10 44.60	1.20 75.10	1.20 35.20			
**Fat mass (kg)**						
Mean (SD)	8.65 (6.49)	11.38 (9.62)	9.23 (8.05) (*N* = 23)			
Median (Q1, Q3)	7.10 (4.50, 10.90)	10.00 (3.40, 17.35)	5.60 (3.65, 14.90)	Kruskal–Wallis	*χ* ^2^ (2) = 0.774	0.679
Range	0.40 31.80	0.20 30.90	0.70 30.80			
**Waist–hip ratio**						
Mean (SD)	0.76 (0.16)	0.77 (0.21)	0.76 (0.15) (*N* = 22)			
Median (Q1, Q3)	0.78 (0.74, 0.83)	0.81 (0.78, 0.88)	0.77 (0.72, 0.86)	Kruskal–Wallis	*χ* ^2^ (2) = 1.838	0.399
Range	0.11 0.95	0.17 0.98	0.23 0.96			
**Cranial perimeter (cm)**						
Mean (SD)	53.93 (3.12) (*N* = 38)	53.87 (2.08)	53.06 (2.55)	ANOVA	F (2, 82) = 0.853	0.43
Median (Q1, Q3)	54.25 (52.00, 56.00)	54.00 (52.70, 55.10)	52.50 (51.00, 55.08)			
Range	47.00 60.00	48.50 57.00	49.00 58.00			
**Abdominal perimeter (cm)**						
Mean (SD)	64.96 (15.90) (*N* = 38)	69.10 (16.06)	64.26 (14.74)	Kruskal–Wallis	*χ* ^2^ (2) = 1.841	0.398
Median (Q1, Q3)	63.00 (55.50, 69.00)	66.00 (58.60, 75.50)	58.50 (54.75, 75.38)			
Range	23.00 114.00	48.00 113.00	44.50 98.00			
**Bicipital skinfold (mm)**						
Mean (SD)	9.77 (5.54)	17.04 (17.95)	11.01 (7.67)			
Median (Q1, Q3)	8.00 (6.50, 11.40)	12.20 (8.60, 18.50)	8.10 (6.00, 14.25)	Kruskal–Wallis	*χ* ^2^ (2) = 6.274	0.043
Range	3.20 32.00	4.00 92.00	4.40 34.20			
**Tricipital skinfold (mm)**						
Mean (SD)	14.55 (5.13)	18.33 (6.50)	15.91 (7.31)			
Median (Q1, Q3)	14.00 (11.20, 17.40)	18.20 (13.40, 20.00)	13.40 (11.00, 19.85)	Kruskal–Wallis	*χ* ^2^ (2) = 5.316	0.07
Range	4.55 28.00	10.00 38.00	7.20 36.20			
**Subscapular skinfold (mm)**						
Mean (SD)	11.32 (7.42)	14.46 (8.70)	13.42 (10.23)			
Median (Q1, Q3)	9.20 (7.00, 11.60)	11.00 (7.90, 20.10)	8.50 (6.00, 19.55)	Kruskal–Wallis	*χ* ^2^ (2) = 2.246	0.325
Range	4.55 37.00	5.00 34.20	2.20 34.00			
**Iliac skinfold (mm)**						
Mean (SD)	12.24 (14.65) (*N* = 38)	14.81 (10.00)	11.54 (8.81)			
Median (Q1, Q3)	8.00 (6.00, 13.45)	12.20 (7.10, 17.10)	7.60 (5.20, 19.00)	Kruskal–Wallis	*χ* ^2^ (2) = 3.045	0.218
Range	3.20 92.00	4.00 33.00	1.00 33.00			
**Abdominal skinfold (mm)**						
Mean (SD)	14.70 (11.45)	17.26 (9.15)	14.46 (8.03)			
Median (Q1, Q3)	12.00 (8.60, 15.00)	15.00 (10.70, 21.50)	13.50 (8.20, 18.65)	Kruskal–Wallis	*χ* ^2^ (2) = 2.609	0.271
Range	3.80 70.00	5.70 36.00	2.00 34.60			
**Total intake (kcal)**						
Mean (SD)	1703.09 (240.13) (*N* = 36)	1829.10 (435.25) (*N* = 20)	1758.30 (497.41) (*N* = 23)			
Median (Q1, Q3)	1668.59 (1586.22, 1784.36)	1880.74 (1580.17, 2010.55)	1625.97 (1415.80, 1994.06)	Kruskal–Wallis	*χ* ^2^ (2) = 2.707	0.258
Range	1255.33 2522.67	822.75 2639.32	1053.96 2903.90			
**Carbohydrate intake (kcal)**						
Mean (SD)	812.72 (210.39) (*N* = 36)	900.78 (280.08) (*N* = 20)	961.03 (305.41) (*N* = 23)			
Median (Q1, Q3)	804.82 (667.04, 899.51)	948.88 (776.54, 1042.95)	911.86 (782.63, 1011.96)	Kruskal–Wallis	*χ* ^2^ (2) = 6.176	0.046
Range	320.35 1462.77	275.95 1352.56	283.34 1662.22			
**Fat intake (kcal)**						
Mean (SD)	613.63 (162.25) (*N* = 36)	651.83 (230.80) (*N* = 20)	581.91 (198.42) (*N* = 23)			
Median (Q1, Q3)	590.13 (497.24, 748.57)	631.62 (459.07, 720.69)	536.49 (465.10, 644.40)	Kruskal–Wallis	*χ* ^2^ (2) = 1.550	0.461
Range	288.00 956.39	321.48 1290.60	314.25 1040.94			
**Protein intake (kcal)**						
Mean (SD)	296.98 (90.60) (*N* = 36)	274.85 (117.84) (*N* = 20)	267.07 (277.49) (*N* = 23)			
Median (Q1, Q3)	279.93 (247.59, 315.95)	270.76 (200.63, 310.31)	198.42 (140.05, 241.89)	Kruskal–Wallis	*χ* ^2^ (2) = 13.591	0.001
Range	181.00 722.83	122.01 607.43	99.99 1391.08			

*Note*: The initial number of patients was 39 Controls, 23 MHPA, and 24 PKU; any differences for individual determinations are detailed in the table.

Abbreviations: ANOVA, analysis of variance (one‐way); BMI, body mass index; MME, musculoskeletal mass.

Next, we evaluated body and BAT temperature in the three experimental groups (Figure 1C–E). Our results showed that while control, MHPA and PKU patients had similar body temperature (Figure 1D), BAT temperature was increased in the PKU group, when compared to controls and MHPA patients (Figure 1C,E). Given that no differences in body temperature were observed, we proceeded to evaluate the gradient Δ(BAT temperature − body temperature) and the ratio of BAT temperature/body temperature. The rationale was that patients who exhibited higher activation of BAT should present larger relationships (delta or ratio) between both magnitudes. In line with the BAT activation in the PKU group, the temperature gradient (Δ(BAT temperature − body temperature); Figure 1F) and the temperature ratio (BAT temperature/body temperature; Figure 1G) were higher or tended to be increased (*p* = 0.053 in the case of temperature ratio) in these patients when compared to controls. Of note, body temperature negatively and highly significantly correlated with both the gradient (Figure 1H) and the ratio (Figure 1I) with the BAT temperature in the three experimental groups (control, MHPA, and PKU patients), demonstrating that lower body temperatures promoted BAT activation in all the patients, independently of their PKU status. Overall, these findings suggested that PKU was associated with a hyperactive BAT thermogenic response, even under apparently normal body temperature conditions.

### 2.2 T4 And FGF21 Are Increased in PKU Patients

**FIGURE 2:**
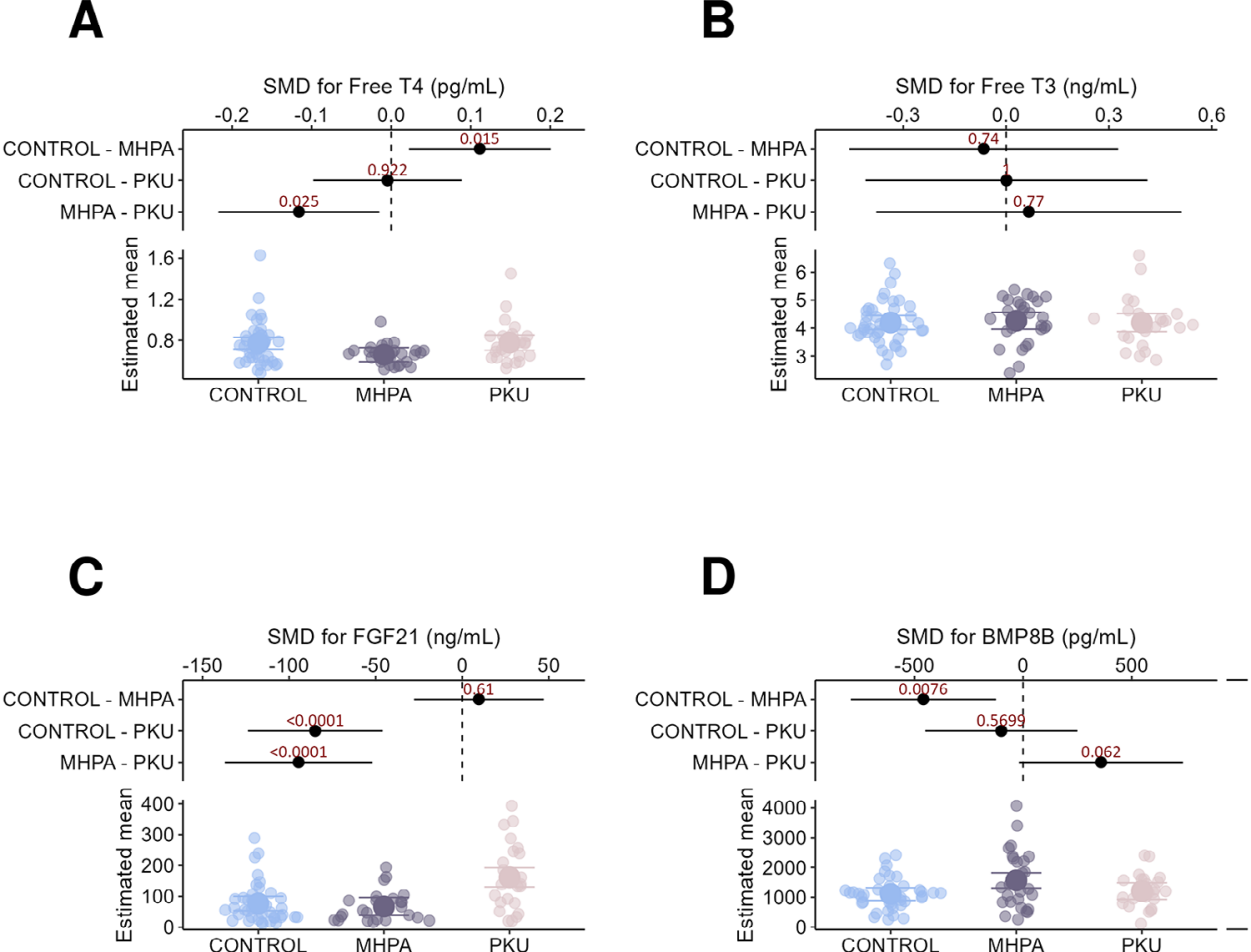
Effect of PKU on circulating thermogenic hormones. (A) Free thyroxine (fT4), (B) free triiodothyronine (fT3), (C) fibroblast growth factor 21 (FGF21), and (D) bone morphogenetic protein 8B (BMP8B) circulating levels in control mild hyperphenylalaninemia (MHPA) and phenylketonuria (PKU) patients. Differences between groups were determined by contrast of marginal means estimated by linear regression. Alpha level was set at 0.05. All statistical tests were two‐sided. SMD, standardized mean difference.

Next, we investigated whether enhanced BAT thermogenesis observed in PKU patients was associated with changes in circulating metabolic parameters and hormones. We analyzed the circulating levels of glucose and lipids. However, no differences were found in the circulating concentrations of glucose (Figure S2A and Table S1), triglycerides (TG; Figure S2B and Table S1), cholesterol (Figure S2C and Table S1), high‐density lipoprotein (HDL; Figure S2D and Table S1), and low‐density lipoprotein (LDL; Figure S2E and Table S1) levels in any of the experimental groups. BAT thermogenesis is regulated by multiple circulating hormones that either act directly on brown adipocytes or influence sympathetic outflow via hypothalamic centers, with THs (T3 and T4), FGF21, and bone morphogenetic protein 8B (BMP8B) among the principal regulators [15, 24, 25, 26]. Therefore, we aimed to investigate if the increased BAT thermogenesis in PKU patients could be associated with elevated levels of some of these hormones (Figure 2A–D). Our results showed increased circulating levels of fT4 (Figure 2A and Table S1; when compared with MHPA patients) and FGF21 (Figure 2C and Table S1), but not fT3 (Figure 2B and Table S1) or BMP8B (Figure 2D and Table S1) in PKU patients. Thyroid‐stimulating hormone (TSH) did not show differences between groups (Table S1). Thus, increased thermogenesis in PKU patients was associated with increased fT4 and/or FGF21 circulating levels.

### 2.3 Body Mass Index Correlated With BAT Temperature in PKU Patients

To further explore the possible link between BAT temperatures with circulating factors, we performed correlation analyses between these parameters. First, to assess whether there could be variables acting as confounders or effect modifiers, the existing association between anthropometric and nutritional variables was calculated, given that if they were associated with both temperature and/or circulating values, this information would have to be considered when estimating the association between them. Our data showed that age, sex, height, musculoskeletal mass (MME), and nutritional parameters (total, carbohydrate, fat, and protein intake) did not show any correlation with either body temperature, BAT temperature, or their gradient of temperatures in PKU patients (Table S2), whereas parameters related with adiposity, such as body weight, fat mass, waist–hip ratio and body mass index (BMI) showed a highly significant negative correlation with BAT temperature (Table S2). Conversely, as expected, in controls (but not in MHPA patients), age and sex were correlated with BAT temperature (Tables S3 and S4). This indicated that the activated thermogenesis in PKU patients was differentially regulated when compared with the other experimental groups.

### 2.4 Tyr, but Not Phe or Catecholamines, Was Associated With Increased Thermogenesis in PKU Patients

Next, a regression analysis was carried out to evaluate the association between BAT temperature and circulating biochemical parameters, as well as the remaining variables. The objective was to determine whether the observed associations differed among groups, specifically controls, MHPA and PKU patients. To evaluate this effect and bearing in mind that the association between BAT temperature and the variable did not have to be linear (it might change along the concentration range), the interaction between them was incorporated into a semiparametric regression model. Importantly, given the dependency on BAT temperature, on BMI (Tables S2–S4), that effect was adjusted in the model:

BATtemperature=Group+BMI+s(Group)Variable

**FIGURE 3:**
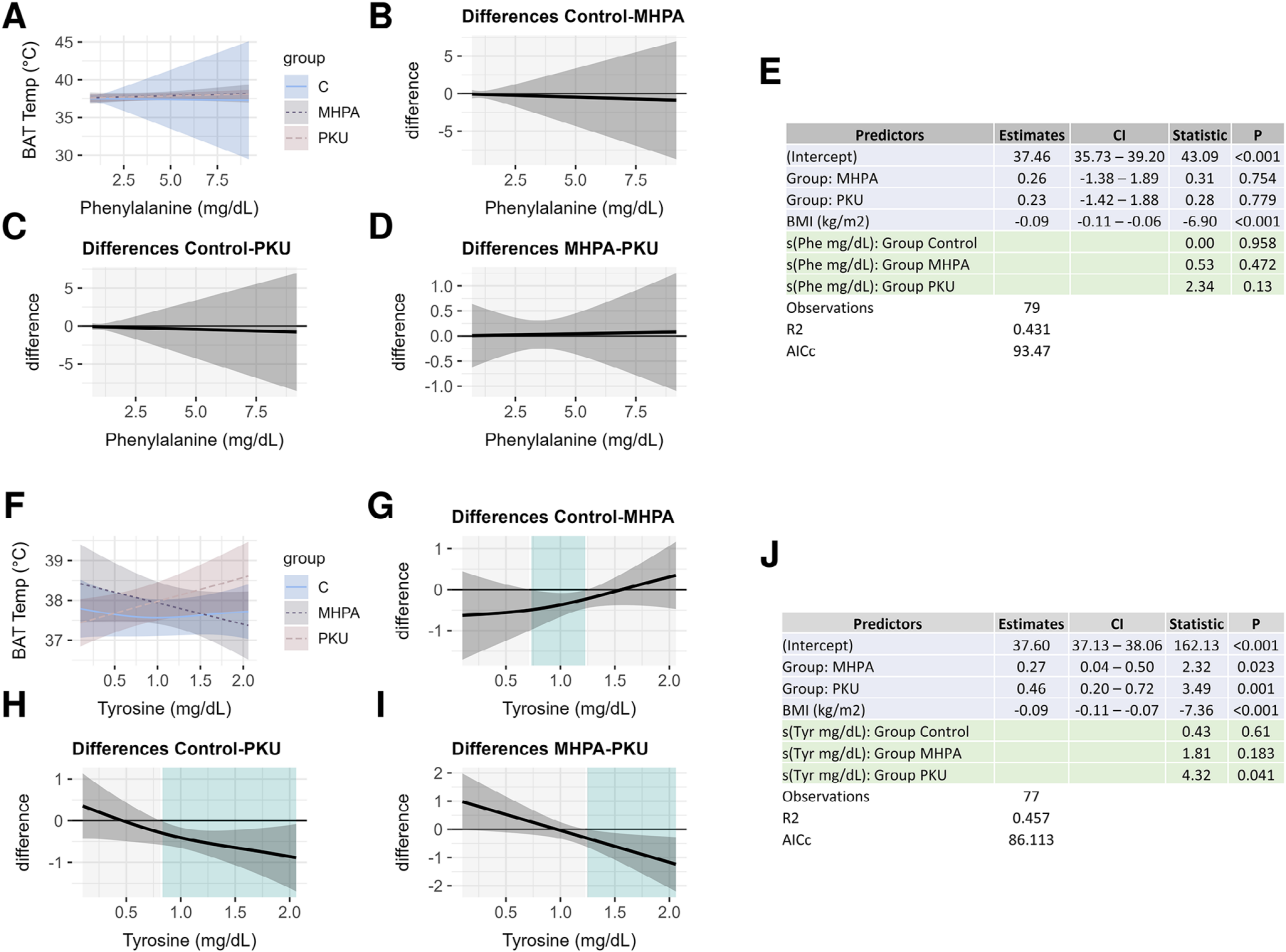
Association of Phe and Tyr with BAT temperature in PKU. (A and F) Estimated smooth effect of phenylalanine (Phe) (A) and tyrosine (Tyr) (F) over brown adipose tissue (BAT) temperature, depending on the group. Mean effect represented as a line (Control [C]: solid blue; mild hyperphenylalaninemia [MHPA]: dotted gray; phenylketonuria [PKU]: dashed red), and 95% confidence interval as shaded band. (B–D and G–I) Differences in effect between Control‐MHPA, Control‐PKU, and MHPA‐PKU groups, shading in green the range of values of Phe or Tyr with significant effect differences between the groups and in gray the range with nonsignificant differences. Horizontal line at 0 is the reference for establishing significance (curve below 0: values of BAT temperature in the first group are lower than in the second group; curve above 0: values of BAT temperature in the first group are higher). (E and J) Semiparametric regression model: (i) Rows 1–4 (in blue) represent the parametric part of the model with its corresponding estimates and confidence intervals (CI); (ii) Rows 5–7 (in green) represent the smooth part of the models with its corresponding statistic and *p* values; and (iii) Rows 8–10 (in white) represent the number of observations, the coefficient of determination (*R* ^2^) and the Akaike information criterion (AICc). Effects are adjusted by body mass index (BMI) given its highly significant effect on BAT temperature. Alpha level was set at 0.05.

**FIGURE 4:**
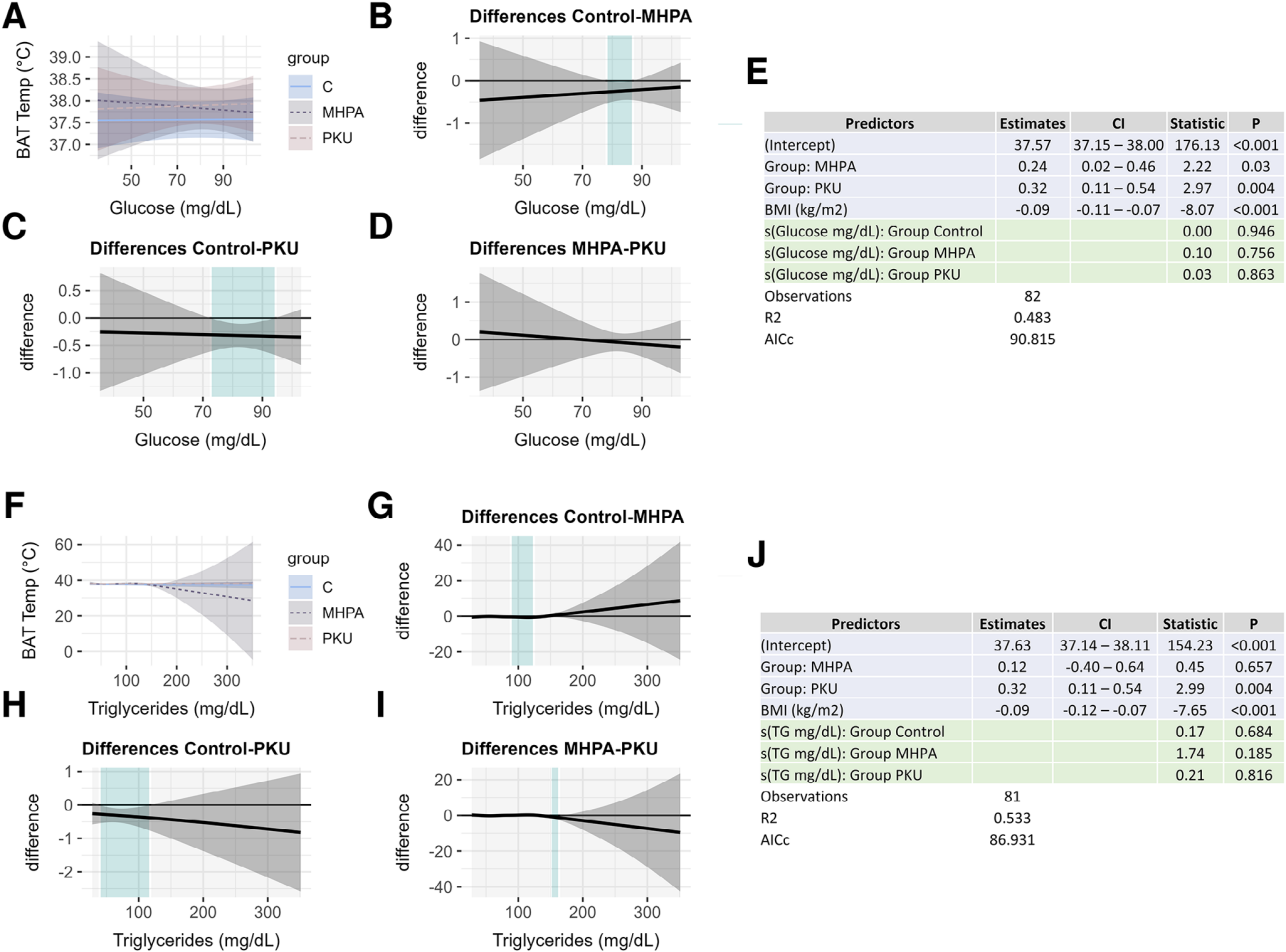
Association of glucose and lipids with BAT temperature in PKU. (A and F) Estimated smooth effect of glucose (A) and triglycerides (TG) (F) over brown adipose tissue (BAT) temperature, depending on the group. Mean effect represented as a line (Control [C]: solid blue; mild hyperphenylalaninemia [MHPA]: dotted gray; phenylketonuria [PKU]: dashed red), and 95% confidence interval as shaded band. (B–D and G–I) Differences in effect between Control‐MHPA, Control‐PKU, and MHPA‐PKU groups, shading in green the range of values of glucose or TG with significant effect differences between the groups and in gray the range with nonsignificant differences. Horizontal line at 0 is the reference for establishing significance (curve below 0: values of BAT temperature in the first group are lower than in the second group; curve above 0: values of BAT temperature in the first group are higher). (E and J) Semiparametric regression model: (i) Rows 1–4 (in blue) represent the parametric part of the model with its corresponding estimates and confidence intervals (CI); (ii) Rows 5–7 (in green) represent the smooth part of the models with its corresponding statistic and *p* values; and (iii) Rows 8–10 (in white) represent the number of observations, the coefficient of determination (*R* ^2^) and the Akaike information criterion (AICc). Effects are adjusted by body mass index (BMI) given its highly significant effect on BAT temperature. Alpha level was set at 0.05.

**FIGURE 5:**
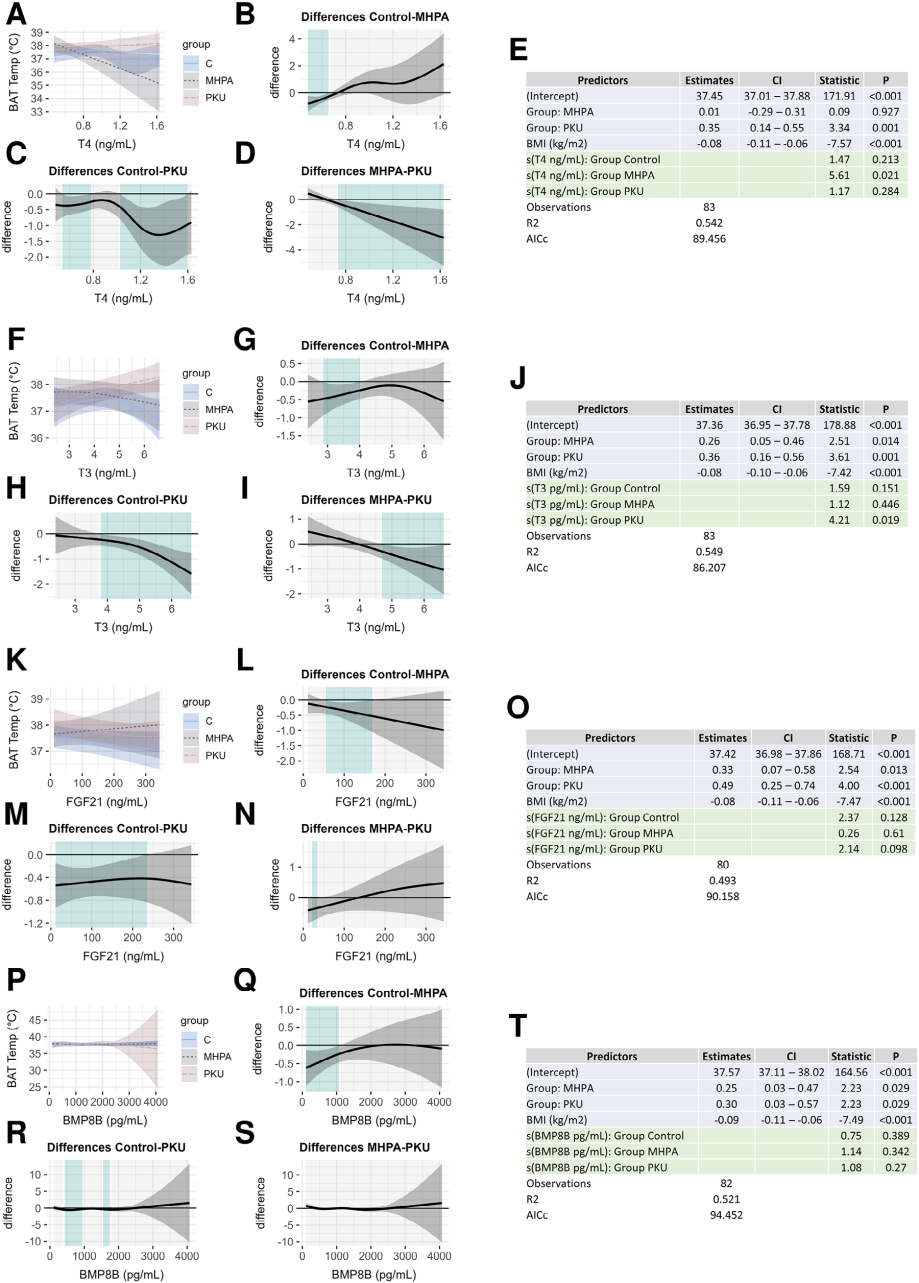
Association of thermogenic hormones with BAT temperature in PKU. (A, F, K, and P) Estimated smooth effect of free thyroxine (fT4) (A), free triiodothyronine (fT3) (F), fibroblast growth factor 21 (FGF21) (K), and bone morphogenetic protein 8B (BMP8B) (P) over brown adipose tissue (BAT) temperature, depending on the group. Mean effect represented as a line (Control [C]: solid blue; mild hyperphenylalaninemia [MHPA]: dotted gray; phenylketonuria [PKU]: dashed red), and 95% confidence interval as shaded band. (B–D, G–I, L–N, and Q–S) Differences in effect between Control‐MHPA, Control‐PKU, and MHPA‐PKU groups, shading in green the range of values of fT4, fT3, FGF21, or BMP8B with significant effect differences between the groups and in gray the range with nonsignificant differences. Horizontal line at 0 is the reference for establishing significance (curve below 0: values of BAT temperature in the first group are lower than in the second group; curve above 0: values of BAT temperature in the first group are higher). (E, J, O, and T) Semiparametric regression model: (i) Rows 1–4 (in blue) represent the parametric part of the model with its corresponding estimates and confidence intervals (CI); (ii) Rows 5–7 (in green) represent the smooth part of the models with its corresponding statistic and *p* values; and (iii) Rows 8–10: represent the number of observations, the coefficient of determination (*R* ^2^) and the Akaike information criterion (AICc). Effects are adjusted by body mass index (BMI) given its highly significant effect on BAT temperature. Alpha level was set at 0.05.

*“Group”* and *“BMI”* represented the main effects of group and BMI, *“(Group)Variable”* represented the interaction between the group and the biochemical parameter (or anthropometric variable), and *“s”* represented the flexible effect of the variable on BAT temperature. Once the model was estimated, a test on the interaction was carried out to assess if there were differences between the three curves (control, MHPA, and PKU). In addition to BMI, other variables, such as age and sex were evaluated, with BMI considered as the most relevant confounder variable due to its higher correlation with BAT temperature (global *P* for the whole cohort of patients = 10^−16^). Despite the BMI having highly significant effect on BAT temperature in all the analyzed variables (Figures 3, 4, 5 and Figure S3), when excluded the effect of the studied variables on BAT temperature was still significant in some cases (i.e., Tyr, fT4, FGF21, and fT3; see below).

Our data showed no association effect of Phe on BAT temperature either at any concentration or the experimental groups (Figure 3A–D), excluding that the levels of this amino acid may have a direct effect to cause BAT activation (Control *p* = 0.958; MHPA *p* = 0.472; PKU *p* = 0.13; Figure 3E). In the case of Tyr, our data showed that in the control group the association was almost flat, but negative in the MHPA patients: when the levels of Tyr increased the temperature of BAT decreased. The opposite effect was found for PKU, where the elevation of Tyr levels was associated with an elevation in BAT temperature (Figure 3F; Control *p* = 0.61; MHPA *p* = 0.183; PKU *p* = 0.041; Figure 3J). When the curves were analyzed, no major differences were found amid control and MHPA (Figure 3G); the most remarkable differences were detected between PKU and the other groups, with BAT temperature for PKU significantly higher as Tyr levels increased. These differences in BAT temperature were significant for Tyr levels above 0.75 and 1.15 mg/dL when compared to controls (Figure 3H) and MHPA group (Figure 3I), respectively. Regression analysis of dopamine (Figure S3A–E), noradrenaline (Figure S3F–J), and adrenaline (Figure S3K–O) demonstrated lack of differences of their effect on BAT temperature among MHPA and PKU patients in all their concentration ranges. Significant differences were found for the lower concentration ranges, with their effect on BAT temperature significantly increased in MHPA and PKU in relation to controls for the three catecholamines. These findings supported a selective role of Tyr in driving BAT activation in this patient population.

### 2.5 Glucose, but Not Triglyceride Levels, Was Associated With Increased Thermogenesis in PKU Patients

While the defense of BAT temperature relies on glucose in early/neonatal human stages [25, 26], lipids are the main source of BAT thermogenesis in more advanced life stages [26, 27]. Given the average age of the patients involved in this study (control: 11.45 ± 0.81 years; MHPA: 10.91 ± 0.84 years; PKU: 10.38 ± 1.03 years), we decided to analyze the involvement of both substrates. Our data showed that the association between glucose and BAT temperature was mostly flat across the three groups (Figure 4A,E; Control *p* = 0.946; MHPA *p* = 0.756; PKU *p* = 0.863) with no statistical differences between MHPA and PKU patients at any concentration (Figure 4D). On the other hand, when both groups were compared with controls, significant differences in the effects on BAT temperature were found in the middle concentration range (Figure 4B,C). Overall, these data suggested that at normoglycemia (75–95 mg/dL) PKU patients were associated with a higher BAT activity. In relation to the circulating levels of TG circulating levels, the regression analysis equally showed that the association between their concentration and BAT temperature was mostly flat in the three groups (Figure 4F,J; Control *p* = 0.684; MHPA *p* = 0.185; PKU *p* = 0.816) with minor statistical differences between control MHPA and PKU patients, only significant in the lower range of concentration (45–120 mg/dL; differences control–PKU) (Figure 4G–I).

### 2.6 THs and FGF21 Correlate With Increased Thermogenesis in PKU Patients

Next, we investigated the possible associations between BAT temperature and endocrine parameters in the three groups. Our data showed that fT4 was not positively associated with BAT temperature in MHPA patients (Figure 5A,E; Control *p* = 0.213; MHPA *p* = 0.021; PKU *p* = 0.284). However, when the difference in the curves was analyzed, PKU patients showed significantly higher values compared to controls and MHPA patients across almost the entire concentration range (Figure 5B–D). When fT3 (the one with higher affinity to the TH receptor, also characterized with a more potent thermogenic effect [28, 29]) was assessed, the results revealed a positive association with BAT temperature in the PKU group (when fT3 increased, BAT temperature increased; Figure 5F,J; Control *p* = 0.151; MHPA *p* = 0.446; PKU *p* = 0.019), but not in control and MHPA patients (for which was negatively associated). In keeping with this data, while the differences in the curves between control and MHPA were only observed in the lower concentration range (Figure 5G), differences with PKU were significant in a broader range (Figure 5H,I). Overall, this evidence suggested that increased BAT thermogenesis in PKU patients was significantly associated with increased levels of THs, when compared with control and MHPA patients. Concentration of circulating FGF21 tended to be negatively associated with BAT temperature in control and PKU patients, but not in the MHPA patients (Figure 5K–O; Control *p* = 0.128; MHPA *p* = 0.61; PKU *p* = 0.098). This might suggest that reduced BAT activity triggered the release of FGF21 as a compensatory mechanism to promote thermogenesis in control and PKU groups. Consistent with this interpretation, curve comparison showed that the regression line for the PKU group was shifted upward relative to controls across almost the entire FGF21 range, whereas no significant differences were detected versus MHPA, indicating a more pronounced FGF21 increase linked to BAT hypoactivation in PKU (Figure 5L–N). By contrast, BMP8B showed no relevant association withg BAT temperature (Figure 5P–T; Control *p* = 0.389; MHPA *p* = 0.342; PKU *p* = 0.27), indicating that this hormone did not contribute to BAT temperature changes in any group (Figure 5Q–S).

### 2.7 Phe Induces FGF21 Expression in Human Hepatocytes and Adipocytes

**FIGURE 6:**
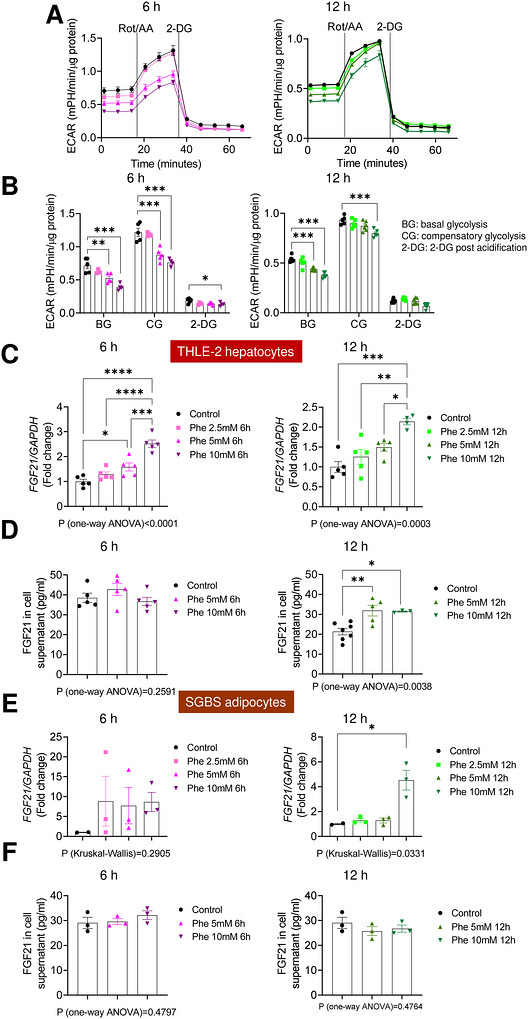
Effect of Phe on human hepatocytes and adipocytes. (A) Real‐time extracellular acidification rate (ECAR), (B) glycolytic parameters (basal [BG] and compensatory [CG] glycolysis and 2‐deoxy‐D‐glucose [2‐DG] post acidification), (C and E) fibroblast growth factor 21 (FGF21; gene name *FGF21*) mRNA levels and (D and F) FGF21 protein levels in the supernatant of transformed human liver epithelial‐2 (THLE‐2) human hepatocytes (A–D) or Simpson–Golabi–Behmel Syndrome (SGBS) human adipocytes (E and F) after phenylalanine (Phe) treatments at different dosages (2.5, 5, and 10 mM) and times (6 and 12 h). Glyceraldehyde‐3‐phosphate dehydrogenase (GAPDH; gene name *GAPDH*) was used as housekeeping gene to serve as an internal control in the mRNA analyses. Data are expressed as mean ± SEM. Statistical significance was determined by one‐way ANOVA followed of Bonferroni test or Kruskal–Wallis followed of Dunn's test; normality was assayed with Shapiro–Wilk test. Alpha level was set at 0.05. ^*^ *p* < 0.05, ^**^ *p* < 0.01, ^***^ *p* < 0.001, and ^****^ *p* < 0.0001. All statistical tests were two‐sided.

FGF21 is a member of fibroblast growth factor family, that is, synthesized mainly in the liver although it is also expressed in adipose tissues, including BAT [30, 31]. FGF21 plays major roles in both thermogenic activation and the adaptive metabolic response to starvation, being induced by changes in amino acid concentration [32]. To investigate whether Phe might induce FGF21 expression, we treated both THLE‐2 (transformed human liver epithelial‐2) human hepatocytes [33, 34] and human SGBS (Simpson–Golabi–Behmel Syndrome) adipocytes [35, 36, 37] with Phe and analyzed the gene expression and secretion to the culture medium of FGF21. The selection of the Phe dosage was done based on former literature about Phe treatments in cell cultures [38, 39, 40] and was chosen to span and exceed levels observed in poorly controlled PKU, allowing detection of cellular responses and exploration of potential threshold or saturation effects. By measuring extracellular acidification rate (ECAR), we found that, as expected [39, 40], Phe decreased the glycolytic rate in THLE‐2 cells in a dose‐dependent and time‐dependent fashion, confirming the efficacy of the treatment, as well as the glycolytic parameters (represented in bars), namely the basal glycolysis, the compensatory glycolysis and the post 2‐deoxyglucose (2‐DG) acidification (Figure 6A,B and Figure S4A,B). We then extended these analyses to mitochondrial parameters and observed that higher Phe doses and longer exposure times induced a significant, dose‑ and time‑dependent reduction in oxygen consumption rate (OCR), ATP production and coupling efficiency (Figure S4C,D), indicating that mitochondrial oxidative phosphorylation is also affected by Phe‑induced metabolic stress.

Phe promoted a very robust stimulating dose‐dependent and time‐dependent effect on *FGF21* mRNA levels after 6 and 12 h (Figure 6C and Figure S4E) and FGF21 secretion to the culture medium (Figure 6D) in human THLE‐2 hepatocytes. The action of Phe on human SGBS adipocytes was milder, just being evident after 12 h of treatment at the highest used dose (10 mM) on *FGF21* mRNA expression (Figure 6E), but not on FGF21 concentration in the cell supernatant (Figure 6F). Expression analyses of other key metabolic genes, such as deiodinase 1 (*DIO1*) in human THLE‐2 hepatocytes (Figure S4F), as well as deiodinase 2 (*DIO2*) (Figure S5A) and uncoupling protein 1 at mRNA (*UCP1*) (Figure S5B) or protein levels (Figure S5C) in human SGBS adipocytes exhibited no changes. Overall, this evidence indicated that the increased FGF21 levels observed in PKU patients were likely of hepatic, rather than BAT, origin.

Recent literature has increasingly highlighted alternative thermogenic pathways that can operate independently of canonical UCP1 activation [41]. To address this possibility, we analyzed the mRNA expression of several markers of those pathways in SGBS adipocytes, including tissue‐nonspecific alkaline phosphatase (TNAP; gene name *ALPL;* creatine kinase futile cycle, Figure S5D), ATP Synthase Subunit e (ATP5K; gene name *ATP5I*; ATP‑dependent futile cycle; Figure S5E), sarco/endoplasmic reticulum Ca^2+^‐ATPase 2b (SERCA2b; gene name *ATP2A2*; calcium‑dependent futile cycle; Figure S5F), and creatine kinase, mitochondrial 1A/B (CKMT1; gene name *CKMT1A/B*; creatine‑kinase futile cycle; Figure S5G). Our data did not reveal any consistent increase in the expression of these markers at any dose or time point. Conversely, *ALPL* (Figure S5D) and *ATP2A2* (Figure S5F) expression were decreased at higher doses and longer incubation times, arguing against the involvement of these pathways in PKU‑induced thermogenesis.

### 2.8 Central Administration of FGF21 to Rodents Increased BAT Thermogenesis

**FIGURE 7:**
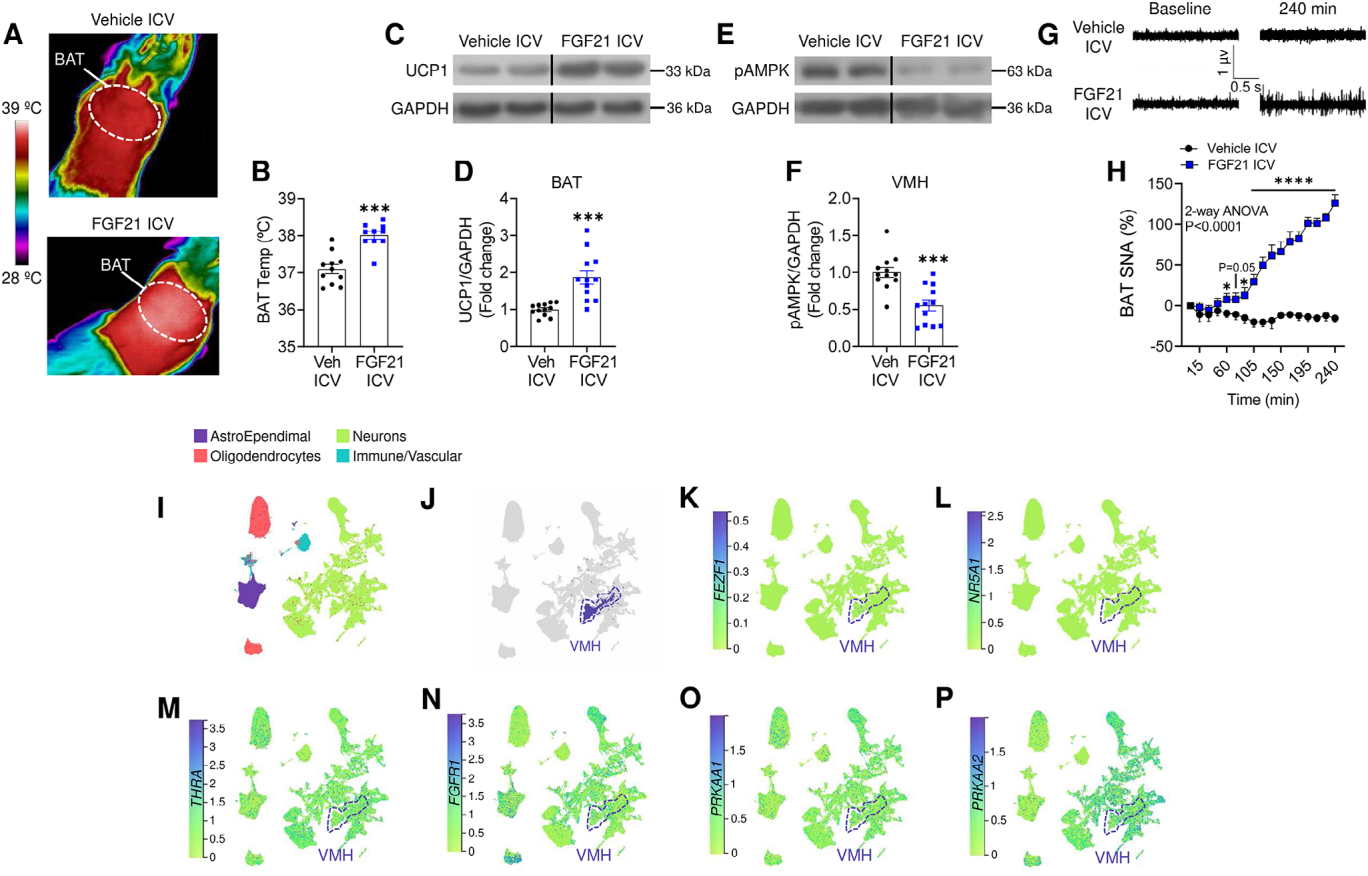
Effect of central FGF21 on BAT thermogenesis, hypothalamic AMPK activity, and signaling system expression in the human hypothalamus. (A) Representative infrared thermographic images of interscapular brown adipose tissue (BAT) from rats after intracerebroventricular (ICV) administration of vehicle or fibroblast growth factor 21 (FGF21). (B) Quantification of BAT surface temperature in vehicle‐ and FGF21‐treated rats. (C and E) Representative immunoblots of uncoupling protein 1 (UCP1) in BAT (C) and phosphorylated AMPK (pAMPK) in the ventromedial nucleus of the hypothalamus (VMH) (E) from vehicle‐ and FGF21‐treated animals; glyceraldehyde‐3‐phosphate dehydrogenase (GAPDH) was used as loading control. (D and F) Densitometric analysis of UCP1 in BAT (D) and pAMPK in VMH (F), expressed as fold change relative to vehicle. (G and H) Representative neurograms (G) and time course (H) of BAT sympathetic nerve activity (SNA) in mice after ICV injection of vehicle or FGF21. (I) Uniform manifold approximation and projection (UMAP) representation of major hypothalamic cell classes from the HypoMap single‐cell RNA‐sequencing dataset [49]. (J) Localization of VMH neuronal clusters (blue outline) within HypoMap. (K and L) Expression of FEZ family zinc finger 1 (FEZF1; gene name *FEZF1*; K) and steroidogenic factor 1 (SF1; gene name *NR5A1*; L) as VMH‐specific markers. (M–P) Expression of thyroid hormone receptor alpha (TRα; gene name *THRA*; M), FGF21 receptor (FGFR1; gene name *FGFR1*; N), AMPKα1 (gene name *PRKAA1*; O), and AMPKα2 (gene name *PRKAA2*; P) in HypoMap, highlighting VMH neuronal populations. In the Western blot analyses, values were expressed in relation to GAPDH. Representative images for all proteins are shown, with all bands for each picture derived from the same gel, although they may be spliced for clarity (vertical black lines). Data are expressed as mean ± SEM. Statistical significance was determined by *t*‐test (B, D, and F) or one‐way ANOVA followed of Bonferroni test (H); normality was assayed with Shapiro–Wilk test. Alpha level was set at 0.05. ^***^ *p* < 0.001 and ^****^ *p* < 0.0001. All statistical tests were two‐sided.

In vitro, Phe failed to induce a thermogenic gene program in SGBS adipocytes, arguing against a direct action of Phe on brown adipocytes. This favored an indirect mechanism, most likely mediated by FGF21 and/or THs acting centrally within the hypothalamus. In this context, AMP‑activated protein kinase (AMPK) in the ventromedial hypothalamus (VMH) is a key mediator of TH (T3/T4, via hypothalamic TRα) actions on BAT thermogenesis [42, 43, 44, 45, 46, 47, 48]. FGF21 has also been implicated in central control of BAT thermogenesis by stimulating sympathetic outflow, including via hypothalamic circuits involving GABAergic neurons in the lateral hypothalamic area (LHA) and zona incerta (ZI). However, whether hypothalamic AMPK participates in the central thermogenic actions of FGF21 has not been previously examined. To directly test this, FGF21 was administered intracerebroventricularly to rats, which increased BAT temperature (Figure 7A,B) and UCP1 expression (Figure 7C,D), reduced AMPK phosphorylation in the VMH (pAMPK; Figure 7E,F), and enhanced BAT sympathetic nerve traffic recorded in mice (Figure 7G,H). Taken together, these results supported the hypothesis that, in PKU, chronically elevated (Phe‑induced) FGF21 and TH levels act within the VMH to inhibit AMPK, thereby increasing sympathetic drive to BAT. This centrally driven sympathetic activation would promote BAT thermogenesis and UCP1 upregulation, providing a plausible mechanistic explanation for the elevated BAT temperature observed in patients.

### 2.9 Central Hypothalamic Signatures of FGF21 and TH Signaling in the Human VMH

In further support of a hypothalamic mechanism in humans, we interrogated the recently published human HypoMap single‐cell atlas of the hypothalamus (https://cellxgene.cziscience.com/) [49]. In this dataset, the human VMH was identified using FEZ family zinc finger 1 (FEZF1; gene name *FEZF1*) and steroidogenic factor 1 (SF1; gene name *NR5A1*) as specific markers (Figure 7I–L). Within this VMH cluster, we observed clear expression of the genes encoding TRα (gene name *THRA*; Figure 7M), the FGF21 receptor (FGFR1; gene name *FGFR1*; Figure 7N), as well as AMPKα1 (gene name *PRKAA1*; Figure 7O) and AMPKα2 (gene name *PRKAA2*; Figure 7P). These observations indicate that certain subpopulations of human VMH neurons are molecularly equipped to sense both THs and FGF21 and to signal through AMPK, providing a translationally relevant substrate for the AMPK(VMH)‐SNS‐BAT axis in humans that we propose in our model. Notably, in the human LHA (Figure S6A,B), FGFR1 (Figure S6C) is also expressed alongside SLC32A1 (solute carrier family 32, GABA vesicular transporter, member 1; gene name *SLC32A1*; Figure S6D) and glutamate decarboxylase 1 (GAD1, the enzyme catalyzing the production of GABA from L‐glutamic acid; gene name *GAD1*; Figure S6E). This transcriptional profile suggested that the FGF21‐responsive‐GABAergic LHA circuit previously described in rodents [50] may also be conserved in humans, providing an additional pathway through which FGF21 could modulate BAT thermogenesis at the hypothalamic level.

## 3 Discussion

PKU is the most common inherited disease of amino acid metabolism, characterized by elevated levels of Phe (and therefore low levels of Tyr), which affects the fetal brain, heart, and nervous system development in the newborn infant, leading to cognitive impairment [1, 2, 3, 4]. Despite the vast knowledge that exists on PKU pathophysiology, data on thermoregulatory mechanism in PKU patients are scarce, despite the long‐standing recognition that they fail to properly defend body temperature after cold exposure [9]. Here, we have investigated BAT thermogenesis using infrared thermography in a cohort of PKU patients in comparison to controls and patients with MHPA, a mild PAH deficiency which does not require treatment. Our data show that at thermoneutrality PKU patients maintain their body temperature in a normal range at the expense of higher BAT temperature and elevated BAT temperature−body temperature gradient and ratio. This suggests that, despite normothermia, the BAT of PKU patients is more activated than controls and individuals with MHPA. One plausible explanation for this apparent paradox is that heat production by BAT is balanced by increased heat dissipation, thereby promoting heat loss and preventing an excessive rise in core temperature. Previous studies in PKU have described abnormalities in thermoregulatory responses, including blunted sweating to heat stimuli, consistent with altered autonomic control [9]. In this context, it is conceivable that increased BAT activation in PKU provides additional heat to maintain normothermia in the face of such autonomic alterations or other metabolic inefficiencies, rather than driving systemic hyperthermia. Our results also showed that BAT activation in PKU patients was associated with body weight, BMI, and fat mass. However, contrary to controls, neither sex, age, height, MME, nor nutritional parameters show any correlation with BAT temperature in PKU patients. This is of importance because, although dietary patterns differ to some extent between groups, these analyses indicate that they are unlikely to be the primary determinant of the BAT thermogenic phenotype observed in PKU. This indicates that BAT function depends on the impact of the disease, and therefore on differential stimuli, in PKU individuals, a hypothesis that we further investigated. BMI was therefore included as the main covariate/confounder in our semiparametric models when analyzing the association between biochemical markers and BAT temperature.

To gain possible mechanistic insight into these results, we analyzed the association between the circulating levels of Phe and Tyr and BAT temperature. Of note, the concentration of Phe (which, as expected [1, 2, 3, 4], was elevated in the PKU patients) did not correlate with brown fat thermogenesis in any of the experimental settings, indicating that this amino acid did not directly impact the activation of BAT in PKU patients. Thus, although it has been described that *N*‐acyl amino acids, such as Phe, are potent mitochondrial uncouplers in human adipocytes [40], this effect does not appear to contribute to the increased BAT thermogenesis observed in PKU patients. However, the lack of correlation between Phe and BAT activation does not exclude the possible (indirect) involvement of this amino acid on thermogenesis. PKU patients display higher circulating levels of *N*‐lactoyl‐phenylalanine (Lac‐Phe, a *N*‐lactoyl‐amino acid, which is a pseudopeptide of lactic acid and Phe) [51, 52]. Current evidence indicates that both lactate and Lac‐Phe exert a marked thermogenic action in rodents [53], opening up the possibility that increased Lac‐Phe in PKU patients may be related to the increased BAT activation, a hypothesis that warrants further investigation. Direct in vitro experiments in human brown or beige adipocytes could test whether exposure to Lac‑Phe Tyr modulates UCP1 expression, mitochondrial respiration or markers of non‑canonical thermogenesis. Such experimental work will be essential to move from the correlative evidence reported here toward a full causal dissection of the molecular pathways linking altered amino acid metabolism and adaptive BAT activation in PKU. Quite opposite to Phe, Tyr levels were positively associated with BAT temperature in the PKU group. The relevance of this observation is intriguing, because it may indicate the involvement of catecholamines in the activation of BAT function in PKU: those patients with higher levels of Tyr would have greater levels of noradrenaline and adrenaline and subsequently, higher BAT tone. However, our results did not reveal any difference either in the plasma concentration of noradrenaline or adrenaline, or in the possible association between those parameters and BAT temperature, excluding a mechanistic link.

Next, we addressed the possible association between BAT temperature and fuel sources, such as glucose and circulating lipids [25, 26]. Analysis of glucose, TG, cholesterol, LDL, and HDL did not reveal any differences between the three experimental groups; however, significant effects on BAT temperature were found among PKU patients relative to controls in the normoglycemic and normolipidemic ranges indicating that likely both glucose and TG contribute to the increased thermogenesis of the PKU patients. This is of importance, because although it is known that the defense of BAT temperature relies on glucose in early/neonatal human stages [25, 26], lipids are the main source of BAT thermogenesis in the adulthood [26, 27]. Thus, our data suggest that in PKU patients at infantile stages, both glucose and lipid account for increased thermogenesis, which may explain the increased activation of BAT, based on a more effective (and abundant) fuel utilization.

We also aimed to address if the increased BAT function in PKU could be associated with elevated levels of some thermogenic endocrine factors, such as fT4, fT3, FGF21, and BMP8B [15, 24, 25, 26]. Our data showed increased circulating levels of FGF21 and fT4 (when compared to MHPA), but neither fT3 nor BMP8B in the PKU group. Intrigued by the higher levels of FGF21 in PKU, we sought to address the underlying molecular mechanism under that effect. Given the multiple factors that preclude performing studies in human patients, especially in pediatric populations, including obvious ethical constraints, we utilized THLE‐2 hepatocytes [33, 34] and human SGBS adipocytes [35, 36, 37] both well‐established human cell models for metabolic studies. Human BAT biopsies, especially in pediatric populations, are scarce, and not suitable for systematic mechanistic work. The SGBS model therefore provides a practical and ethical human in vitro system that reproduces key features of adipocyte browning [35, 36, 37]. Our data demonstrated that Phe markedly increased the expression at both mRNA and protein levels of FGF21 in human hepatic cells, whereas its effect on human adipocytes was weaker. This evidence may indicate that the increased FGF21 circulating levels in PKU patients have a hepatic, rather than adipocytic, source. In addition, these results elicit two relevant questions: (i) whether the increase of Phe has a direct effect on gene expression of *Fgf21* and (ii) what the molecular mechanism is. Regulation of gene expression by amino acids is mediated through several mechanisms affecting both the DNA transcription and mRNA translation. It is known that Phe regulates the feeding‐induced synthesis and secretion of pancreatic enzymes, such as α‐amylase, trypsin, and lipase, through mRNA translation initiation factors, such as mechanistic target of rapamycin (mTOR), ribosomal protein S6 kinase beta‐1 (S6K1), and eukaryotic translation initiation factor 4E‐binding protein 1 (4EBP1) [54, 55]. Therefore, it would be tempting to speculate that a similar mechanism might be involved in the induction of hepatic FGF21 expression by Phe.

When analyzing the possible association between increased BAT function and the hormonal milieu of PKU patients, our results demonstrated that the increased thermogenesis in PKU patients exhibited a positive correlation with fT3, the active form of THs [28, 29], indicating that increases in fT3 were associated with enhanced brown fat activation in these patients. To gain further insight, we evaluated the potential association between BAT temperature and the circulating levels of FGF21 and BMP8B. For BMP8B, we did not detect any association/differential effect with BAT temperature in any of the groups. This result was not unexpected, as although BMP8B plays a major role in the regulation of neonatal thermogenesis [25, 26], studies have shown that its circulating levels are high at birth, but decline progressively and do not correlate with BAT temperature in the later in childhood [56]. Regarding FGF21, our data showed its negative association (and notably, differentially with controls) with BAT temperature in the PKU patients, suggesting that BAT hypoactivation highly elicits the release of FGF21 to promote thermogenesis.

The lack of a detectable thermogenic response to Phe in our in vitro adipocyte model prompted us to consider that the key mechanism linking PKU to BAT activation is unlikely to be adipocyte‑autonomous, but rather centrally mediated. Central FGF21 was sufficient to recapitulate fundamental features of the PKU thermogenic phenotype, namely an increase in BAT temperature and upregulation of UCP1 in BAT. Crucially, these effects were accompanied by inhibition of AMPK in the VMH and a marked rise in sympathetic nerve activity (SNA) directed to BAT, mirroring the well‑established mechanism through which THs stimulate thermogenesis via the AMPK(VMH)‐SNS‐BAT axis [42, 43, 44, 45, 46, 47, 48]. Although additional peripheral or adipocyte‑intrinsic contributions cannot be excluded, the convergence of negative in vitro results for Phe, central FGF21/T3 interventions in rodents, and human hypothalamic transcriptomic data strongly supports a scenario in which inhibition of VMH AMPK and consequent enhancement of sympathetic outflow to BAT constitute a key mechanistic link between the PKU endocrine milieu and the sustained BAT thermogenesis documented in our cohort.

Our study has several limitations that should be acknowledged. (i) First, the cohort consists predominantly of children and adolescents, which is relevant given the strong age‑ and sex‑dependent variation in BAT prevalence and activity described in pediatric populations [17, 19, 20]. Dedicated studies in adult PKU patients will be required to determine whether the thermogenic phenotype we report is preserved or modified later in life. (ii) Second, although we provide converging clinical and experimental evidence implicating the AMPK(VMH)‐SNS‐BAT axis and hormones such as FGF21 and T3/T4, further mechanistic work using targeted gain‐ and loss‐of‐function approaches (receptor blockade or gene‑silencing) is needed to formally establish causality and dissect tissue‑specific contributions. (iii) Finally, several metabolic and environmental determinants of BAT activation were only partially captured: detailed physical activity, ambient temperature exposure, pre‐measurement meal timing, scan time‐of‐day, and season. In this sense, although 3‐day food diaries reflected stable PKU dietary regimens and measurements occurred under standardized hospital conditions, these clinical practice limitations should be addressed in future controlled studies. Thus, longitudinal cohorts incorporating these factors will be important to fully characterize the complexity of BAT regulation in PKU and related metabolic disorders.

## 4 Conclusion

Overall, this study shows that increased BAT activation in pediatric PKU patients is associated with altered THs and FGF21 signaling as part of the PKU phenotype, but not with BMP8B, and is accompanied by reduced hypothalamic AMPK activity and enhanced sympathetic drive to BAT. Together with single‐cell RNA‐sequencing data indicating that AMPK, TRα, and FGF21 receptor pathways converge in specific hypothalamic neuronal populations, these findings support a hypothalamic, AMPK‑dependent mechanism linking disrupted amino acid metabolism to altered energy balance in PKU. This integrated evidence refines our understanding of the whole‐body phenotype of pediatric PKU patients and may inform future strategies for its clinical management.

## 5 Materials and Methods

### 5.1 Patients

A cohort of 86 Caucasian patients was recruited (Table S1) at the Clinical University Hospital of Santiago de Compostela (CHUS; Santiago de Compostela, Galicia, Spain) and University Hospital Lucus Augusti (HULA; Lugo, Galicia, Spain). The age‐ and sex‐matched control group consisted of healthy participants. Inclusion criteria were a diagnosis of MHPA or PKU due to PAH deficiency confirmed by genetic testing. Metabolic control and dietary adherence were evaluated from median blood Phe levels and predefined age‐specific “safe” thresholds, as previously described [57]. Exclusion criteria were any additional disease that could affect BAT function, non‐adherence to inclusion criteria, or absence of written informed consent. Data points were only excluded when insufficient serum volume was available for a given assay.

### 5.2 Feeding Questionnaires

For each participant, dietary intake was assessed using a 3‑day food record. A trained dietitian reviewed the records with patients and/or caregivers and converted reported foods into daily energy and macronutrient intake (kcal from carbohydrates, fats, and proteins; Table 1). Average intake over the 3‑day period was calculated using ODIMET software (www.odimet.es; Unit of Diagnosis and Treatment of Congenital Metabolic Diseases, CHUS, Santiago de Compostela, Spain).

### 5.3 Anthropometric Analyses

Body weight was measured to the nearest 0.1 kg with patients wearing light clothing and no shoes, using an electronic medical scale (Seca 701; Seca GmbH & Co. KG, Hamburg, Germany). Height was measured barefoot with a Harpenden stadiometer. BMI was calculated as weight divided by height squared (kg/m^2^). Body composition was assessed by multi‑frequency (5, 50, 250, and 500 kHz) bioelectrical impedance using an 8‑electrode hand‑to‑foot analyzer (InBody230; InBody Co. Ltd., Seoul, South Korea) in the standing position. This system was chosen because it is child‑friendly, rapid, and feasible in our pediatric outpatient setting, where supine research‑grade assessments were not practical. All measurements were obtained under strictly standardized conditions with the same device and protocol (barefoot standing on the electrode panel, hand electrodes held with arms relaxed, avoiding skin‑to‑skin contact), so any known systematic bias of upright InBody devices in absolute fat and fat‑free mass is expected to affect groups similarly and not compromise between‑group comparisons. The procedure lasted ∼60 s and yielded body weight MME and fat mass.

### 5.4 Animals

Adult male Sprague–Dawley rats (8–10 weeks, 200–250 g; CEBEGA‐USC, Santiago de Compostela, Spain) and C57BL/6J mice (9–11 weeks, 27–30 g; University of Iowa, Iowa City, IA, USA) were used. Animals were housed under a 12‑h light/dark cycle at 21°C–24°C and 55% humidity, with ad libitum access to standard chow (Teklad Global 18% Protein Rodent Diet, 2918; Envigo RMS Inc., Indianapolis, IN, USA) and tap water. Group sizes were based on previous studies and 3Rs considerations, with random allocation to experimental groups and duplicate experiments. Handling and cage placement were alternated to minimize systematic housing or measurement bias.

### 5.5 Temperature Measurements

A digital thermometer (Termo Digital C202; Terumo Europe N.V., Leuven, Belgium; 0.1°C scale, ±0.2°C accuracy) was used to measure peripheral body temperature at the right armpit. BAT temperature was recorded with an infrared camera (E60bx: Compact‐Infrared‐Thermal‐Imaging‐Camera; FLIR Systems, Inc., Wilsonville, OR, USA). In relation to the emissivity (*ε*) and considering the age range of the patients [25, 58, 59], it was set at 0.95, as shown [25]. The analyses were also done with other *ε* values in the human skin range (0.91–0.99) [25, 58, 59] and the obtained data/correlations were similar, and the conclusions of the study were the same [25]. For the BAT temperature analysis, the supraclavicular fossa area was selected [27, 41, 60]. Focus distance was set at the interval 40–50 cm based on our former work [25]. The size of that area and the landmarks were similar for all the people of the same age range. Room temperature was set at 22°C–23°C. In rats, the interscapular area was selected, as shown [61, 62, 63, 64]. The analysis of the images was performed with the FLIR Tools Software Package (FLIR) [25, 61, 62, 63, 64].

### 5.6 Blood Extraction

Blood samples were obtained by venipuncture in the lower forearm at 09:00 h after overnight fasting and thermography. A total of 9 mL of blood was collected: 3 mL for routine analyses, 3 mL for catecholamine measurements, and 3 mL for the remaining assays.

### 5.7 Blood Biochemistry

Circulating glucose and lipids were measured by the GPO‑Trinder method (ADVIA 2400 Chemistry System; Siemens Healthineers, Tarrytown, NY, USA). Plasma amino acids and catecholamines were quantified by HPLC. Plasma hormones were measured using ELISA kits: fT3 (EIA3801; DRG Instrumental GmbH, Marburg, Germany), fT4 (EIA3775; DRG Instrumental GmbH, Marburg, Germany), FGF21 (ab222506; Abcam plc, Cambridge, UK), and BMP8B (MBS944757; MyBioSource, Inc., San Diego, CA, USA), as shown [25]. All samples were analyzed in duplicate.

### 5.8 Cell Culture and In Vitro Treatments

THLE‐2 cells (CRL‐2706; RRID: CVCL_3803; ATCC, Manassas, VA, USA) were cultured in BEBM supplemented with BEGM BulletKit (Lonza Walkersville, Inc., Walkersville, MD, USA), 70 ng/mL phosphoethanolamine, 5 ng/mL epidermal growth factor, 10% FBS, and 1% glutamine‐penicillin‐streptomycin (Merck KGaA, Darmstadt, Germany). THLE‐2 cells were treated with 2.5, 5, or 10 mM L‐Phe [38, 39, 40] (Pharmpur GmbH, Königsbrunn, Germany) for 1, 6, or 12 h. Human SGBS preadipocytes (RRID: CVCL_GS28) were cultured in DMEM/F12 with 10% FBS. Once confluent, they were differentiated into adipocytes using a serum‐free adipogenic medium (QuickDiff Differentiation Kit; Lonza Walkersville, Inc., Walkersville, MD, USA) followed by a lipogenic medium (3FC) [35, 36, 37]. Fully differentiated SGBS adipocytes were then treated with 2.5, 5, or 10 mM L‐Phe [38, 39, 40] for 1, 6, or 12 h.

### 5.9 Central Treatments in Rodents

Lateral intracerebroventricular (ICV) cannulae were stereotaxically implanted under ketamine/xylazine anesthesia, as described [42, 44, 45, 46, 47, 48, 63, 64]. After recovery, rats and mice received a single ICV administration of FGF21 (Sigma‐Aldrich, St. Louis, MO, USA) for 3 h (rats) or 4 h (mice), or vehicle (saline).

### 5.10 Sympathetic Nerve Activity Recording

Multi‐fiber recording of SNA was obtained from the nerve subserving BAT, as reported before in mice [42, 45, 65]. Each mouse was anaesthetized and then equipped for direct multifiber SNA from the nerves serving the sub‐scapular BAT. Under a stable isothermal (37.5°C) condition and anesthesia, baseline BAT SNA was recorded over a 10‐min period. Next, FGF21 (1 µg; Sigma‐Aldrich, St. Louis, MO, USA) or vehicle (2 µL) was injected ICV and SNA recorded for an additional 4 h. At the end of the experiment, the background noise was subtracted to measure real SNA, by recording the activity remaining after death.

### 5.11 Analysis of Mitochondrial Respiration and Glycolytic Activity

Mitochondrial respiration and glycolytic activity were assessed as OCR and ECAR, respectively, (Seahorse‐XFe96; Agilent Technologies, Inc., Santa Clara, CA, USA), as shown [66]. Cells were seeded at 1.5 × 10^4^ cells/well and exposed to L‐phenylalanine (Pharmpur GmbH, Königsbrunn, Germany) in dose‐response (2.5, 5, 10 mM) and time‐course (1, 6, 12 h) protocols. Basal OCR and ECAR were recorded in five consecutive measurements. Glycolytic function was evaluated with the Glycolytic Rate Assay (Agilent Technologies, Inc., Santa Clara, CA, USA) by sequential addition of rotenone/antimycin A followed by 50 mM 2‐DG. Mitochondrial respiration was assessed (Cell Mito Stress Test; Agilent Technologies, Inc., Santa Clara, CA, USA) by sequential injection of oligomycin (1.5 µM), FCCP (1 µM), and rotenone/antimycin‐A (0.5 µM). Glycolytic parameters (basal glycolysis, compensatory glycolysis, post‐2‐DG acidification) and mitochondrial parameters (basal, ATP‐linked and maximal respiration, proton leak, spare respiratory capacity, non‐mitochondrial OCR) were calculated accordingly. All OCR and ECAR values were normalized to total protein content per well.

### 5.12 qRT‐PCR Analysis

Total RNA was isolated with TRIzol (Thermo Fisher Scientific Inc., Waltham, MA, USA). In THLE‐2 cells, qRT‐PCR was performed with SYBR Green (Agilent Technologies, Inc., Santa Clara, CA, USA) and the following primers (Eurofins Genomics GmbH, Ebersberg, Germany): *DIO1*, forward 5′‐CCAGAACAGCACGAACTTCCTC‐3′, reverse 5′‐CACTGCCTGAGAGGCTCTACAT‐3′; *FGF21*, forward 5′‐ACTCCAGTCCTCTCCTGCAA‐3′, reverse 5′‐GCACAGGAACCTGGATGTCT‐3′; *GAPDH*, forward 5′‐GTCTCCTCTGACTTCAACAGC‐3′, reverse 5′‐ACCACCCTGTTGCTGTAGCCAA‐3′. Gene expression was normalized to GAPDH and analyzed by the 2^−^ΔΔCt method; all samples were run in duplicate and averaged. In SGBS cells, qRT‐PCR was performed using validated TaqMan assays (Thermo Fisher Scientific Inc., Waltham, MA, USA) for *ALPL* (Hs01029144_m1), *ATP5I* (Hs00273015_m1), *ATP2A2* (Hs01564013_m1), *CKMT1AB* (Hs00179727_m1), *DIO2* (Hs00255341_m1), *FGF21* (Hs00173927_m1), *RPLP0* (Hs99999902_m1), and *UCP1* (Hs00222453_m1).

### 5.13 Western Blotting

Protein lysates from BAT and hypothalamus were separated by SDS‐PAGE, electrotransferred onto PVDF membranes (Merck Millipore, Darmstadt, Germany), and probed with antibodies against UCP1 (1:10,000, ab10983, RRID: AB_2241462; Abcam plc, Cambridge, UK), pAMPK (Thr172; 1:1000, 2535S, RRID: AB_331250; Cell Signaling Technology, Inc., Danvers, MA, USA), and GAPDH (1:5 000, ABS16, RRID: AB_11212362; Merck Millipore, Darmstadt, Germany), as described previously [42, 44, 45, 46, 47, 48, 65]. Immunoreactive bands were visualized on autoradiographic film (Fujifilm Corporation, Tokyo, Japan), scanned, and quantified by densitometry using ImageJ 1.44 (National Institutes of Health, Bethesda, MD, USA), and values were expressed relative to GAPDH.

### 5.14 ELISA Analysis

FGF21 in the supernatant of cells was determined using commercial kits (for THLE‐2: FGF‐21 Human ELISA Kit [ab125966; Abcam plc, Cambridge, UK]; for SGBS: Human ELISA Kit [RD191108200R; BioVendor R&D, Brno, Czech Republic]).

### 5.15 Single‐Cell RNA‐Sequencing Analysis

Single‐cell RNA‐seq data were obtained from the human HypoMap dataset [49], and visualization was performed using the online browser (https://cellxgene.cziscience.com/). Using the Author Categories “C0_named” cell‐type annotation, we selected the major cell‐type subclusters, comprising 63,111 AstroEpendymal cells, 175,109 Oligodendrocytes, 166,475 Neurons, and 28,674 Immune/Vascular cells. Using the Author Categories “region” annotation, we identified the VMH and LHA subclusters, containing 9009 and 22,325 cells, respectively. Relative expression of *FEZF1*, *NR5A1*, *THRA*, *FGFR1*, *PRKAA1*, *PRKAA2*, *SLC32A1*, and *GAD1* was visualized as color‐coded expression maps, highlighting VMH and/or LHA subcluster areas.

### 5.16 Statistical Analysis

Data are presented as mean ± SEM. Analyses were conducted using GraphPad Prism 8.0.2 (GraphPad Software, San Diego, CA, USA) and the lme4 [67], emmeans [68], and mgcv [69] packages in R (R Core Team, Vienna, Austria). Comparisons (two‐sided) were performed using one‐way ANOVA or Kruskal–Wallis tests for anthropometric, nutritional, and in vitro variables, and Student's *t*‐tests for in vivo data. Associations between continuous variables were assessed with Pearson's or Spearman's correlation coefficients, as appropriate. Relationships between continuous outcomes and categorical factors (e.g., sex, study group) were evaluated using unpaired *t*‐tests or one‐way ANOVA, with non‐parametric alternatives (Mann–Whitney *U* or Kruskal–Wallis) when normality and homoscedasticity were violated. Group differences in temperature and circulating parameters were analyzed by linear regression with contrasts of estimated marginal means. Potential nonlinear associations between temperature and biochemical variables were examined using generalized additive models (GAMs) [70] with group‐specific smooth terms; between‐group differences were assessed by curve differentiation.

## Author Contributions

N.L.‐R. and P.S.‐P. recruited patients, collected anthropometric and temperature data, and collected blood samples. A.E.‐S. and P.F.‐S. performed blood biochemistry. P.F.‐S. performed the Western blot analysis. A.C., M.P., F.V., and R.N. performed the in vitro analyses. V.F., C.F., and L.M.S. performed the central administration experiments. D.A.M. and K.R. performed the sympathetic nervous system activity experiments. N.L.‐R., M.P.P., A.E.‐S., C.D., I.G.G., A.U., and M.L. analyzed the human data. M.P.P. and M.L. performed the statistical analyses. N.L.‐R., A.U., C.D., M.L.C., and M.L. designed the human protocols. All authors discussed and interpreted the data. M.P.P. and M.L. made the figures. M.L. developed the hypothesis, secured funding, supervised and directed the project, and wrote the manuscript. All authors have read and approved the final manuscript.

## Funding

The research leading to these results has received funding from the Ministerio de Ciencia e Innovación, co‐funded by the FEDER Program of EU (FV: PID2023‐146781OB‐I00; RN: PID2021‐126096NB‐I00; ML: PID2024‐162486OB‐I00, CPP2024‐011411, and PDC2025‐166594‐I00); the European Research Council (RN: ERC Synergy Grant‐2019‐WATCH‐810331); the US National Institutes of Health (KR: R01 HL162773 and R01 HL172944); and the US Department of Veterans Affairs (KR: I01 BX004249 and IK6 BX006040). The funders had no role in the study design, data collection and analysis, decision to publish, or preparation of the manuscript.

## Ethics Statement

The study in patients was approved by the Ethical Committee and the Committee for Clinical Investigation of the CHUS and HULA (code 2019/147). The study design and conduct complied with all relevant regulations regarding the use of human study participants and were conducted in accordance with the criteria set by the Declaration of Helsinki. Written parental permission was obtained for all patients, and written assent was additionally obtained for those aged 12 years and older. The experiments in rodents were conducted in accordance with international laws on animal experimentation, with guidelines set by the National Institutes of Health, and were approved by the USC Ethical Committee (Project ID 15012/2020/010) and the University of Iowa Animal Care and Use Committee (Protocol 8101549). Animal experiments were conducted in accordance with the 3R principles (Replacement, Reduction, and Refinement). For the single‑cell RNA‑sequencing analyses using publicly available datasets, we confirm that only de‑identified, openly accessible data were used in accordance with the original studies’ ethical approvals and data‑sharing policies; no additional ethical approval or consent from our institutions was required for the reuse of these datasets.

## Conflicts of Interest

Author Miguel López is an Editorial Board member of MedComm. Author Miguel López was not involved in the journal's review of or decisions related to this manuscript. Author Miguel López is an inventor of patents related to hypothalamic AMPK targeting for the treatment of obesity and associated comorbidities: EP21382763, PCT/EP2022/071463; EP24382172.5, PCT/EP2025/054384; and EP24382790.4, PCT/EP2025/070615. Author Miguel López serves as Scientific Director of Gazella Biotech (https://gazellabiotech.com/) and Lyrea Biotech. Author Rubén Nogueiras serves on the Advisory Board of Albor Biotech (https://alborbiotech.com/). The remaining authors declare no conflicts of interest.

## Supporting information




**Table S1**. Circulating parameters of participants
**Table S2**. Correlations between temperature and anthropometric and nutritional parameters in PKU patients
**Table S3**. Correlations between temperature and anthropometric and nutritional parameters in controls
**Table S4**. Correlations between temperature and anthropometric and nutritional parameters in MHPA patients
**FIGURE S1**. Effect of PKU on circulating catecholamines
**FIGURE S2**. Effect of PKU on circulating glucose and lipids
**FIGURE S3**. Association of catecholamines with BAT temperature in PKU
**FIGURE S4**. Effect of Phe on human hepatocytes
**FIGURE S5**. Effect of Phe on human adipocytes
**FIGURE S6**. Expression of GABAergic and FGF21 receptor markers in the human lateral hypothalamic area

## Data Availability

Data generated or analyzed during the study are available from the corresponding author upon request.
