# Supplementary material for: Increased Brown Adipose Tissue Thermogenesis in Phenylketonuria
Source: MedComm (2020). 2026 Jun 13;7(6):e70820. doi: 10.1002/mco2.70820 (PMC13263790; doi:10.1002/mco2.70820)
Supplement: Supplementary file 1 — Table S1. Circulating parameters of participants Table S2. Correlations between temperature and anthropometric and nutritional parameters in PKU patients Table S3. Correlations between temperature and anthropometric and nutritional parameters in controls Table S4. Correlations between temperature and anthropometric and nutritional parameters in MHPA patients FIGURE S1. Effect of PKU on circulating catecholamines FIGURE S2. Effect of PKU on circulating glucose and lipids FIGURE S3. Association of catecholamines with BAT temperature in PKU FIGURE S4. Effect of Phe on human hepatocytes FIGURE S5. Effect of Phe on human adipocytes FIGURE S6. Expression of GABAergic and FGF21 receptor markers in the human lateral hypothalamic area [file MCO2-7-e70820-s001.pdf]

Increased brown adipose tissue thermogenesis in phenylketonuria

Noemí López-Rey [1,2,3], Alba Cabaleiro [1,4], María P. Pata [5], Marion Peyrou [4,6], Ánxela Estévez-Salguero [1,4], Paola Fernández-Sanmartín [1,4], Donald A. Morgan [7], Vitor Ferreira [1,4], Paula Sánchez-Pintos [2], Cintia Folgueira [1,4], Carlos Diéguez [1,4], Adela Urisarri [2], Ismael González-García [1,4,8], Luisa M. Seoane [4,9], Kamal Rahmouni [7,10], Francesc Villarroya [4,6], María L. Couce [2,11], Rubén Nogueiras [1,4] & Miguel López [1,4,*]

[1] Department of Physiology, CIMUS, University of Santiago de Compostela, Santiago de Compostela, Spain
[2] Department of Pediatrics, Neonatology Service, University Clinical Hospital of Santiago de Compostela, Instituto de Investigación Sanitaria de Santiago de Compostela (IDIS) and Spanish Network in Maternal, Neonatal, Child and Developmental Health Research (RICORS-SAMID), Santiago de Compostela, Spain
[3] Research Unit, Hospital Lucus Augusti, Lugo, Spain
[4] CIBER Fisiopatología de la Obesidad y Nutrición (CIBEROBN), Santiago de Compostela, Spain
[5] Biostatech Advice, Training and Innovation in Biostatistics, S.L., Ames, Spain
[6] Departament de Bioquímica i Biomedicina Molecular, Institut de Biomedicina de la, Universitat de Barcelona (IBUB), and Institut de Recerca Hospital Sant Joan de Déu, Barcelona, Spain
[7] Department of Neuroscience and Pharmacology, University of Iowa, Iowa City, Iowa, USA
[8] Neuroendocrine Regulation of Metabolism Group, Instituto de Investigación Sanitaria de Santiago de Compostela (IDIS), Santiago de Compostela, Spain
[9] Grupo Fisiopatología Endocrina, Área de Endocrinología, Instituto de Investigación Sanitaria de Santiago de Compostela (IDIS), Santiago de Compostela, Spain
[10] Veterans Affairs Health Care System, Iowa City, Iowa, USA
[11] CIBER Enfermedades Raras (CIBERER), Travesía Choupana, Santiago de Compostela, Spain

Noemí López-Rey, Alba Cabaleiro, and María P. Pata contributed equally to this work and are joint first authors.

*: **Corresponding author:**
Miguel López; Email: m.lopez@usc.es
Department of Physiology, CIMUS, University of Santiago de Compostela, Santiago de Compostela, 15782, Spain
CIBER Fisiopatología de la Obesidad y Nutrición (CIBEROBN), Santiago de Compostela, 15706, Spain

Short title: BAT thermogenesis in phenylketonuria

Table S1. Circulating parameters of participants

	Control (N=39)	MHPA (N=23)	PKU (N=24)	Test	Statistical	P
**Phenylalanine (mg/dL)**						
N	36	23	24			
Mean (SD)	0.92 (0.15)	3.38 (1.22)	5.56 (3.30)	Welch ANOVA	F (2,30)= 68.315	<0.001
Median (Q1, Q3)	0.88 (0.80, 1.00)	3.29 (2.71, 4.00)	4.41 (3.10, 7.08)			
Range	0.70 - 1.21	0.99 - 6.96	1.60 - 16.25			
**Tyrosine (mg/dL)**						
N	35	22	23			
Mean (SD)	1.23 (0.34)	1.18 (0.23)	0.96 (0.50)			
Median (Q1, Q3)	1.14 (1.08, 1.40)	1.18 (1.07, 1.26)	0.81 (0.70, 1.13)	Kruskal-Wallis	$\chi 2$ (2) = 13.132	0.001
Range	0.10 - 2.06	0.73 - 1.63	0.47 - 2.80			
**Alanine (mg/dL)**						
N	36	23	22			
Mean (SD)	2.71 (0.78)	2.78 (0.84)	2.98 (0.84)	ANOVA	F (2, 78) = 0.764	0.469
Median (Q1, Q3)	2.79 (2.30, 3.14)	2.60 (2.33, 3.23)	2.97 (2.40, 3.39)			
Range	0.58 - 4.42	0.65 - 4.66	1.44 - 4.87			
**Metionine (mg/dL)**						
N	36	23	24			
Mean (SD)	0.35 (0.08)	0.32 (0.05)	0.26 (0.08)	Welch ANOVA	F (2, 51) = 9.446	<0.001
Median (Q1, Q3)	0.32 (0.28, 0.40)	0.32 (0.28, 0.34)	0.24 (0.21, 0.31)			
Range	0.19 - 0.51	0.25 - 0.47	0.10 - 0.43			
**Dopamine (pg/mL)**						
N	32	23	21			
Mean (SD)	18.12 (9.65)	18.78 (9.02)	24.19 (21.93)			
Median (Q1, Q3)	15.00 (10.00, 26.25)	17.00 (11.00, 25.00)	17.00 (12.00, 23.00)	Kruskal-Wallis	$\chi 2$ (2) = 0.866	0.649
Range	8.00 - 42.00	9.00 - 41.00	10.00 - 93.00			
**Noradrenaline (pg/mL)**						

N	32	23	22			
Mean (SD)	256.34 (145.91)	251.04 (143.00)	375.73 (317.95)			
Median (Q1, Q3)	245.00 (136.00, 336.00)	214.00 (148.00, 339.50)	319.00 (118.75, 456.00)	Kruskal-Wallis	χ2 (2) = 0.890	0.641
Range	20.00 - 635.00	67.00 - 617.00	43.00 - 1185.00			
**Adrenaline (pg/mL)**						
N	33	23	21			
Mean (SD)	28.36 (12.31)	29.57 (11.93)	22.90 (12.48)			
Median (Q1, Q3)	25.00 (21.00, 35.00)	29.00 (20.50, 36.00)	18.00 (14.00, 28.00)	Kruskal-Wallis	χ2 (2) = 5.406	0.067
Range	11.00 - 67.00	12.00 - 56.00	10.00 - 59.00			
**Ratio Dopamine/Tyrosine**						
N	28	22	20			
Mean (SD)	1.45e-06 (9.05e-07)	1.67e-06 (8.12e-07)	3.00e-06 (2.80e-06)			
Median (Q1, Q3)	1.11e-06 (7.60e-07, 1.80e-06)	1.58e-06 (1.00e-06, 1.91e-06)	1.99e-06 (1.61e-06, 2.98e-06)	Kruskal-Wallis	χ2 (2) = 7.498	0.024
Range	4.37e-07 - 3.37e-06	5.88e-07 - 3.46e-06	3.93e-07 - 1.19e-05			
**Ratio Noradrenaline/Tyrosine**						
N	29	22	21			
Mean (SD)	2.19e-05 (1.32e-05)	2.35e-05 (1.80e-05)	4.65e-05 (4.04e-05)			
Median (Q1, Q3)	2.00e-05 (1.30e-05, 2.95e-05)	1.83e-05 (1.37e-05, 2.77e-05)	3.89e-05 (1.36e-05, 6.30e-05)	Kruskal-Wallis	χ2 (2) = 4.054	0.132
Range	1.39e-06 - 5.79e-05	5.54e-06 - 8.45e-05	3.93e-06 - 1.46e-04			
**Ratio Adrenaline/Tyrosine**						
N	29	22	20			
Mean (SD)	2.33e-06 (1.32e-05)	2.65e-06 (1.32e-06)	3.02e-06 (2.36e-06)			
Median (Q1, Q3)	2.30e-06 (1.71e-06, 2.75e-06)	2.28e-06 (1.57e-06, 3.85e-06)	2.32e-06 (1.40e-06, 3.75e-06)	Kruskal-Wallis	χ2 (2) = 0.390	0.823
Range	5.88e-07 - 4.84e-06	1.02e-06 - 5.00e-06	3.57e-07 - 8.31e-06			
**Free T4 (ng/mL)**						
N	39	23	24			
Mean (SD)	0.77 (0.22)	0.64 (0.07)	0.78 (0.20)			
Median (Q1, Q3)	0.75 (0.61, 0.85)	0.66 (0.57, 0.69)	0.75 (0.65, 0.84)	Kruskal-Wallis	χ2 (2) = 10.049	0.007

Range	0.48 - 1.63	0.52 - 0.78	0.53 - 1.45			
**Free T3 (pg/mL)**						
N	39	23	24			
Mean (SD)	4.21 (0.78)	4.16 (0.82)	4.20 (0.89)	ANOVA	F (2, 83) = 0.030	0.97
Median (Q1, Q3)	4.03 (3.77, 4.67)	4.15 (3.67, 4.68)	4.17 (3.71, 4.52)			
Range	2.71 - 6.32	2.39 - 5.38	2.86 - 6.61			
**TSH (mU/L)**						
N	29	13	8			
Mean (SD)	1.90 (0.77)	2.20 (1.21)	1.68 (0.99)			
Median (Q1, Q3)	1.80 (1.36, 2.45)	1.73 (1.41, 2.03)	1.40 (1.20, 2.03)	Kruskal-Wallis	χ2 (2) = 1.141	0.565
Range	0.49 - 3.36	1.15 - 4.52	0.43 - 3.43			
**FGF21 (ng/mL)**						
N	38	23	23			
Mean (SD)	78.97 (64.97)	69.19 (59.58)	161.77 (109.68)			
Median (Q1, Q3)	60.22 (32.79, 94.52)	54.66 (24.55, 83.73)	138.28 (79.54, 242.89)	Kruskal-Wallis	χ2 (2) = 12.593	0.002
Range	11.33 - 289.25	16.33 - 269.51	20.08 - 394.33			
**BPM8B (pg/mL)**						
N	38	23	24			
Mean (SD)	1050.79 (452.81)	1365.27 (900.73)	1205.52 (555.00)	Welch ANOVA	F (2, 42) = 1.556	0.223
Median (Q1, Q3)	1070.89 (809.50, 1231.12)	1186.25 (737.70, 1716.31)	1162.44 (902.00, 1578.95)			
Range	253.46 - 2295.95	253.59 - 4070.34	110.18 - 2405.46			
**Glucose (mg/dL)**						
N	38	23	24			
Mean (SD)	85.43 (11.63)	83.35 (6.36)	82.21 (9.51)			
Median (Q1, Q3)	87.50 (80.50, 91.75)	83.00 (79.00, 87.00)	82.50 (77.00, 85.25)	Kruskal-Wallis	χ2 (2) = 4.436	0.109
Range	35.40 - 103.00	70.00 - 95.00	56.00 - 104.00			
**Triglycerides (mg/dL)**						
N	37	23	23			

Mean (SD)	64.73 (22.36)	72.04 (30.54)	87.13 (69.71)			
Median (Q1, Q3)	63.00 (46.00, 80.00)	64.00 (49.00, 90.00)	63.00 (50.00, 91.50)	Kruskal-Wallis	χ2 (2) = 0.958	0.619
Range	31.00 - 140.00	38.00 - 151.00	28.00 - 351.00			
**Cholesterol (mg/dL)**						
N	38	23	23			
Mean (SD)	161.11 (30.50)	152.78 (21.25)	153.09 (24.04)			
Median (Q1, Q3)	158.00 (141.25, 173.25)	156.00 (142.00, 164.00)	151.00 (137.00, 172.00)	Kruskal-Wallis	χ2 (2) = 1.025	0.599
Range	101.00 - 263.00	112.00 - 205.00	101.00 - 195.00			
**HDL (mg/dL)**						
N	35	21	23			
Mean (SD)	53.03 (10.87)	54.67 (17.27)	51.70 (11.87)			
Median (Q1, Q3)	51.00 (45.00, 61.00)	49.00 (45.00, 58.00)	47.00 (44.00, 62.00)	Kruskal-Wallis	χ2 (2) = 0.531	0.767
Range	32.00 - 77.00	35.00 - 114.00	35.00 - 74.00			
**LDL (mg/dL)**						
N	35	21	23			
Mean (SD)	95.97 (28.41)	85.76 (17.63)	84.30 (20.09)			
Median (Q1, Q3)	89.00 (81.50, 109.00)	87.00 (76.00, 99.00)	90.00 (71.50, 96.50)	Kruskal-Wallis	χ2 (2) = 2.454	0.293
Range	40.00 - 188.00	49.00 - 112.00	46.00 - 122.00			

Abbreviations (alphabetical order)

ANOVA: analysis of variance (one way)
BMP8B: bone morphogenetic protein 8B)
Free T3: free triiodothyronine
Free T4: free thyroxine
FGF21: fibroblast growth factor 21
HDL: high-density lipoprotein
MHPA: mild hyperphenylalaninemia
LDL: low-density lipoprotein
PKU: phenylketonuria
Q1: first quartile (25th percentile)
Q3: third quartile (75th percentile)
SD: standard deviation
TSH: thyroid-stimulating hormone
Welch ANOVA: Welch's analysis of variance

Table S2. Correlations between temperature and anthropometric and nutritional parameters in PKU patients

	Body Temp (ºC)		BAT Temp (ºC)		ΔTBAT-Tbody	
	r	P	r	P	r	P
**Age (years)**	-0.051	0.813	-0.422	0.05	0.288	0.182
**Sex**	-0.175	0.413	-0.36	0.1	0.052	0.812
**Height (cm)**	-0.008	0.971	-0.327	0.137	0.205	0.349
**Body weight (kg)**	-0.075	0.727	-0.477	0.025	0.186	0.396
**BMI (kg/m^2)**	-0.201	0.346	-0.66	<0.001	0.178	0.417
**MME (kg)**	0.098	0.657	-0.197	0.392	0.326	0.139
**Fat mass (kg)**	-0.359	0.092	-0.707	0.001	-0.226	0.312
**Waist–hip ratio**	-0.038	0.867	-0.572	0.007	0.242	0.291
**Cranial perimeter (cm)**	-0.185	0.388	-0.384	0.077	0.028	0.898
**Abdominal perimeter (cm)**	-0.256	0.227	-0.623	0.002	0.155	0.481
**Bicipital skinfold (mm)**	-0.093	0.666	-0.586	0.004	0.152	0.49
**Tricipital skinfold (mm)**	-0.083	0.699	-0.587	0.004	0.274	0.205
**Subscapular skinfold (mm)**	-0.108	0.617	-0.624	0.002	0.309	0.151
**Iliac skinfold (mm)**	-0.202	0.343	-0.647	0.001	0.126	0.566
**Abdominal skinfold (mm)**	-0.273	0.196	-0.627	0.002	0.073	0.742
**Total intake (kcal)**	-0.108	0.623	-0.117	0.614	0.095	0.673
**Carbohydrate intake (kcal)**	-0.161	0.464	-0.278	0.223	0.162	0.471
**Fat intake (kcal)**	-0.379	0.075	-0.061	0.793	-0.296	0.18
**Protein intake (kcal)**	-0.103	0.639	-0.071	0.758	0.064	0.778

Abbreviations (alphabetical order)
BMI: body mass index
MME: musculoskeletal mass

Table S3. Correlations between temperature and anthropometric and nutritional parameters in controls

	Body Temp (ºC)		BAT Temp (ºC)		ΔTBAT-Tbody	
	**r**	**P**	**r**	**P**	**r**	**P**
**Age (years)**	-0.108	0.514	-0.33	0.04	0.173	0.293
**Sex**	-0.106	0.508	0.449	0.003	-0.496	0.001
**Height (cm)**	-0.127	0.428	-0.274	0.082	0.207	0.207
**Body weight (kg)**	-0.043	0.789	-0.493	0.001	0.283	0.081
**BMI (kg/m^2)**	-0.023	0.888	-0.752	<0.001	0.57	<0.001
**MME (kg)**	-0.005	0.974	-0.394	0.013	0.266	0.102
**Fat mass (kg)**	-0.174	0.29	-0.668	<0.001	0.389	0.014
**Waist–hip ratio**	-0.024	0.884	-0.737	<0.001	0.515	0.001
**Cranial perimeter (cm)**	-0.078	0.633	-0.292	0.067	0.302	0.066
**Abdominal perimeter (cm)**	-0.259	0.107	-0.46	0.003	0.292	0.075
**Bicipital skinfold (mm)**	0.12	0.456	-0.418	0.007	0.494	0.001
**Tricipital skinfold (mm)**	-0.058	0.72	-0.36	0.021	0.268	0.1
**Subscapular skinfold (mm)**	0.01	0.949	-0.486	0.001	0.419	0.008
**Iliac skinfold (mm)**	0.017	0.918	-0.585	<0.001	0.434	0.006
**Abdominal skinfold (mm)**	-0.034	0.835	-0.371	0.018	0.281	0.083
**Total intake (kcal)**	-0.033	0.846	-0.032	0.852	-0.033	0.847
**Carbohydrate intake (kcal)**	0.193	0.252	-0.149	0.378	0.224	0.189
**Fat intake (kcal)**	-0.029	0.865	-0.023	0.891	-0.076	0.66
**Protein intake (kcal)**	0.039	0.82	0.105	0.538	-0.039	0.822

Abbreviations (alphabetical order)
BMI: body mass index
MME: musculoskeletal mass

Table S4. Correlations between temperature and anthropometric and nutritional parameters in MHPA patients

	Body Temp (ºC)		BAT Temp (ºC)		ΔTBAT-Tbody	
	r	P	r	P	r	P
**Age (years)**	0.031	0.887	0.025	0.911	0.175	0.426
**Sex**	-0.393	0.039	0.333	0.089	-0.56	0.005
**Height (cm)**	-0.163	0.406	0.19	0.342	0.065	0.769
**Body weight (kg)**	0.101	0.608	-0.072	0.721	0.468	0.024
**BMI (kg/m^2)**	0.155	0.442	-0.389	0.049	0.623	0.001
**MME (kg)**	-0.064	0.772	0.04	0.859	0.099	0.652
**Fat mass (kg)**	0.396	0.062	-0.428	0.047	0.542	0.008
**Waist–hip ratio**	0.305	0.157	-0.376	0.085	0.546	0.007
**Cranial perimeter (cm)**	-0.012	0.95	0.084	0.678	0.225	0.303
**Abdominal perimeter (cm)**	0.193	0.326	-0.293	0.138	0.656	0.001
**Bicipital skinfold (mm)**	0.245	0.219	-0.312	0.121	0.585	0.003
**Tricipital skinfold (mm)**	0.189	0.345	-0.391	0.048	0.612	0.002
**Subscapular skinfold (mm)**	0.279	0.158	-0.509	0.008	0.789	<0.001
**Iliac skinfold (mm)**	0.313	0.112	-0.456	0.019	0.649	0.001
**Abdominal skinfold (mm)**	0.365	0.072	-0.6	0.002	0.739	<0.001
**Total intake (kcal)**	-0.066	0.76	0.052	0.814	0.138	0.563
**Carbohydrate intake (kcal)**	-0.049	0.821	0.153	0.487	-0.023	0.925
**Fat intake (kcal)**	-0.192	0.368	0.116	0.599	<0.001	1
**Protein intake (kcal)**	0.211	0.322	-0.042	0.849	0.54	0.014

Abbreviations (alphabetical order)
BMI: body mass index
MME: musculoskeletal mass

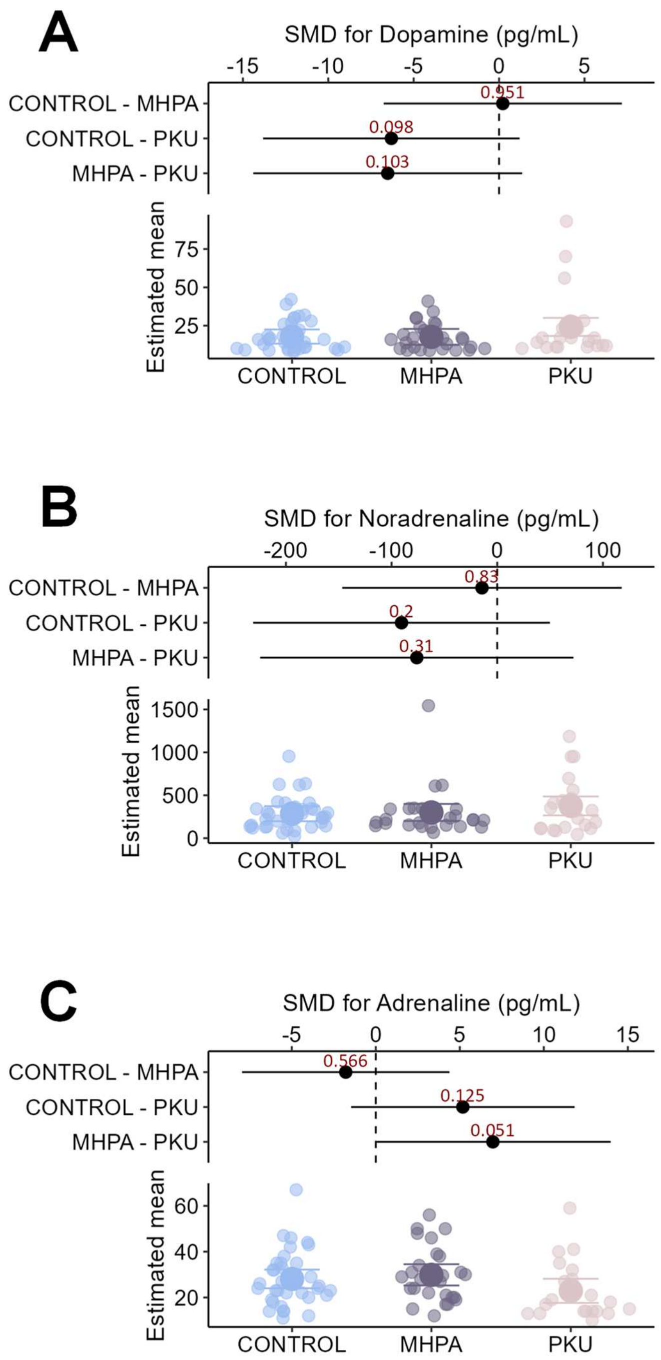

FIGURE S1. Effect of PKU on circulating catecholamines
(A) Dopamine, **(B)** noradrenaline and **(C)** adrenaline circulating levels in control, mild hyperphenylalaninemia (MHPA) and phenylketonuria (PKU) patients. Differences between groups were determined by contrast of marginal means estimated by linear regression. Statistical significance was determined by One-way ANOVA or Kruskal-Wallis and contrast difference of means. Alpha level was set at 0.05. All statistical tests were two-sided. SMD: standardized mean difference.

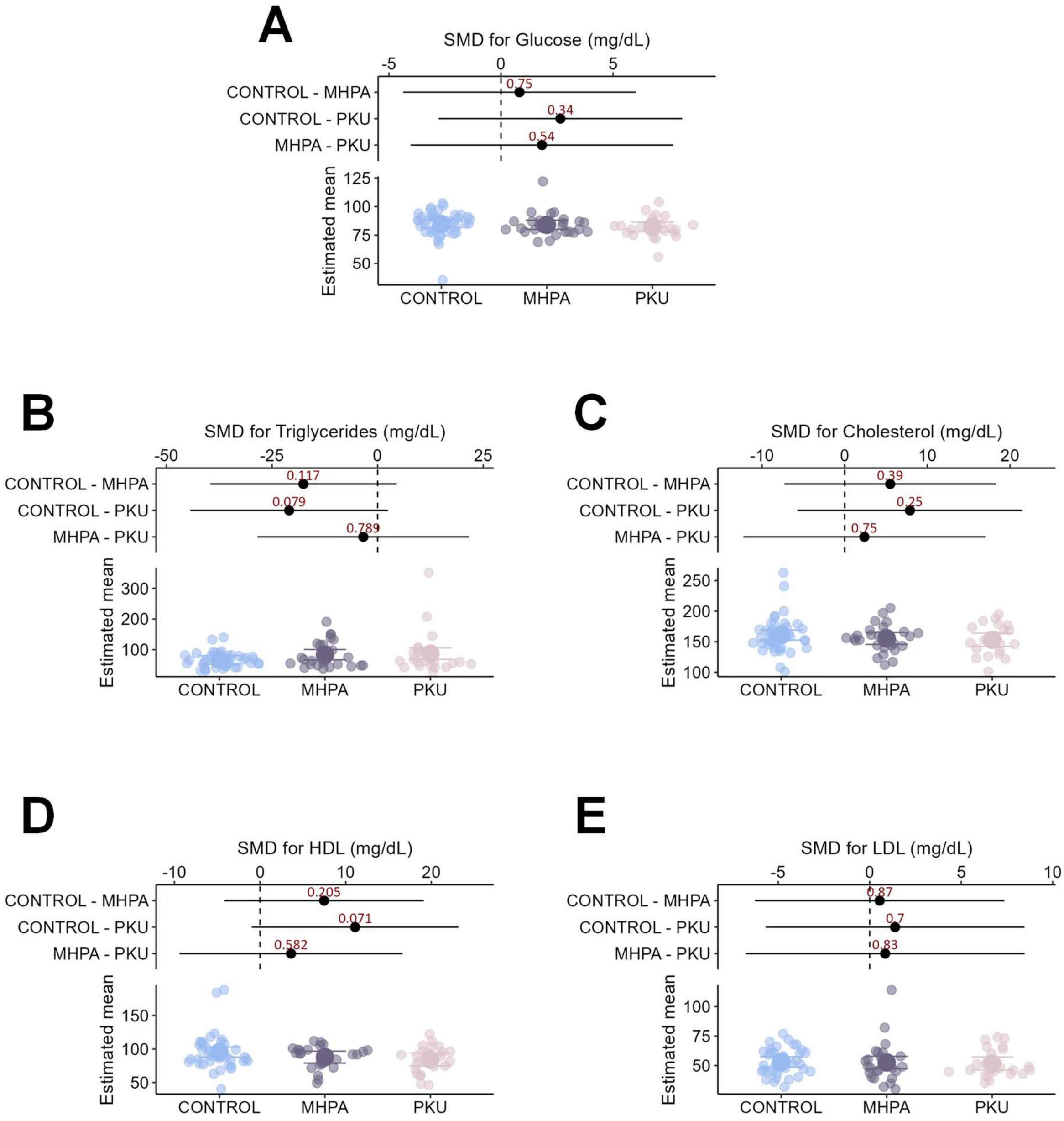

FIGURE S2. Effect of PKU on circulating glucose and lipids

(A) Glucose, **(B)** triglycerides, **(C)** cholesterol, **(D)** high-density lipoprotein (HDL) and **(E)** low-density lipoprotein (LDL) circulating levels in control, mild hyperphenylalaninemia (MHPA) and phenylketonuria (PKU) patients. Differences between groups were determined by contrast of marginal means estimated by linear regression. Alpha level was set at 0.05. All statistical tests were two-sided. SMD: standardized mean difference.

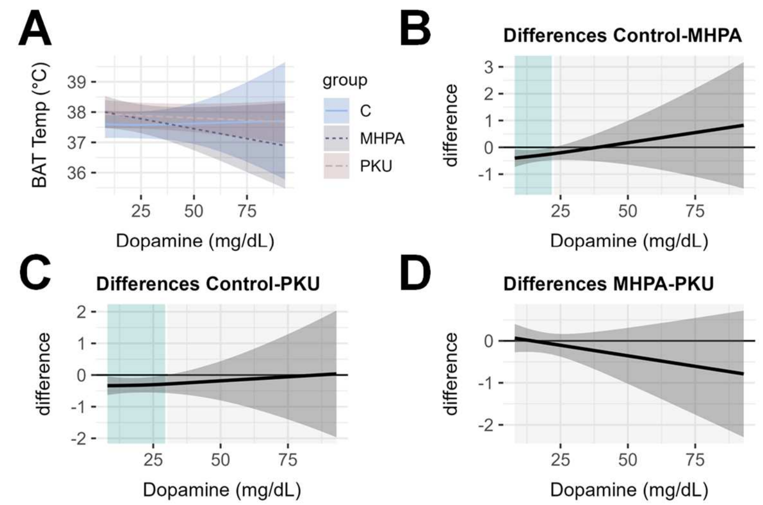

E

Predictors	Estimates	CI	Statistic	P
(Intercept)	37.59	37.15 – 38.03	170.80	<0.001
Group: MHPA	0.26	0.04 – 0.48	2.34	0.023
Group: PKU	0.31	0.08 – 0.54	2.69	0.009
BMI (kg/m2)	-0.09	-0.11 – -0.07	-8.26	<0.001
s(Dopamine pg/mL): Group Control			0.02	0.968
s(Dopamine pg/mL): Group MHPA			1.92	0.17
s(Dopamine pg/mL): Group PKU			0.52	0.475
Observations	73			
R2	0.506			
AICc	80.433			

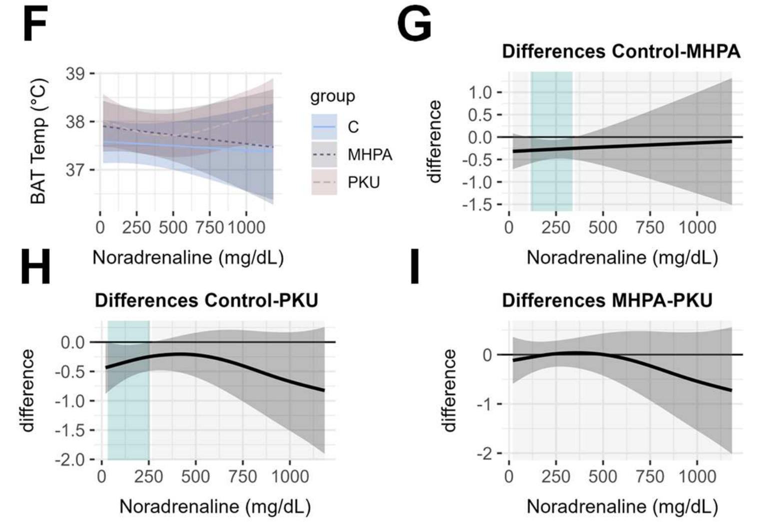

J

Predictors	Estimates	CI	Statistic	P
(Intercept)	37.54	37.11 – 37.97	173.69	<0.001
Group: MHPA	0.26	0.05 – 0.48	2.44	0.017
Group: PKU	0.30	0.07 – 0.52	2.64	0.01
BMI (kg/m2)	-0.09	-0.11 – -0.07	-8.22	<0.001
s(Noradrenaline pg/mL): Group Control			0.15	0.699
s(Noradrenaline pg/mL): Group MHPA			0.41	0.52
s(Noradrenaline pg/mL): Group PKU			1.42	0.316
Observations	75			
R2	0.527			
AICc	80.373			

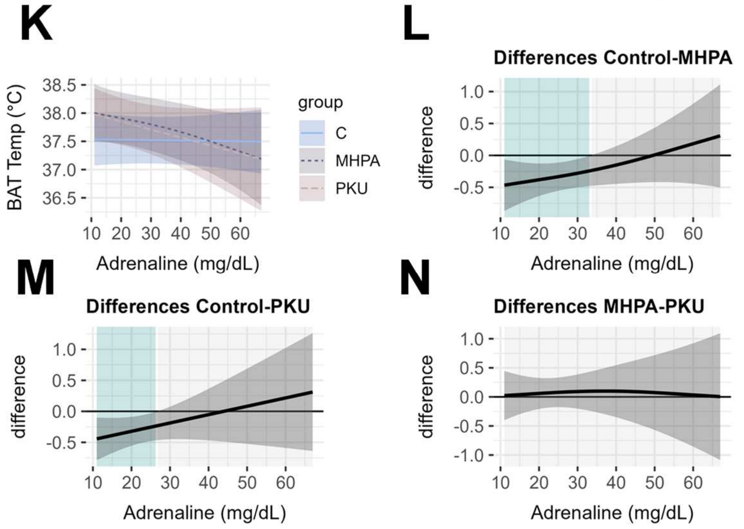

O

Predictors	Estimates	CI	Statistic	P
(Intercept)	37.52	37.10 – 37.95	176.93	<0.001
Group: MHPA	0.30	0.09 – 0.51	2.81	0.007
Group: PKU	0.23	-0.01 – 0.47	1.92	0.059
BMI (kg/m2)	-0.09	-0.11 – -0.07	-8.23	<0.001
s(Adrenaline pg/mL): Group Control			0.02	0.898
s(Adrenaline pg/mL): Group MHPA			3.23	0.094
s(Adrenaline pg/mL): Group PKU			2.29	0.135
Observations	74			
R2	0.537			
AICc	76.503			

FIGURE S3. Association of catecholamines with BAT temperature in PKU
(A, F and K) Estimated smooth effect of dopamine **(A)** noradrenaline **(F)** and adrenaline **(K)** over BAT temperature, depending on the group. Mean effect represented as a line [Control (C): solid blue; mild hyperphenylalaninemia (MHPA): dotted grey; phenylketonuria (PKU) dashed red], and 95% confidence interval as shaded band. **(B-D, G-I and L-N)** differences in effect between Control-MHPA, Control-PKU and MHPA-PKU groups, shading in green the range of values of dopamine, noradrenaline and adrenaline with significant effect differences between the groups and in grey the range with non-significant differences. Horizontal line at 0 is the reference for establishing significance (**curve below 0:** values of BAT temperature in the first group are lower than in the second group; **curve above 0**: values of BAT temperature in the first group are higher). **(E, J and O)** Semi-parametric regression model: **(i) rows 1-4 (in blue)** represent the parametric part of the model with its corresponding estimates and confidence intervals (CI); **(ii) rows 5-7 (in green)** represent the smooth part of the models with its corresponding statistic and P values. **(iii) rows 8-10:** represent the number of observations, the coefficient of determination (R^2) and the Akaike information criterion (AICc). Effects are adjusted by body mass index (BMI) given its highly significant effect on BAT temperature. Alpha level was set at 0.05.

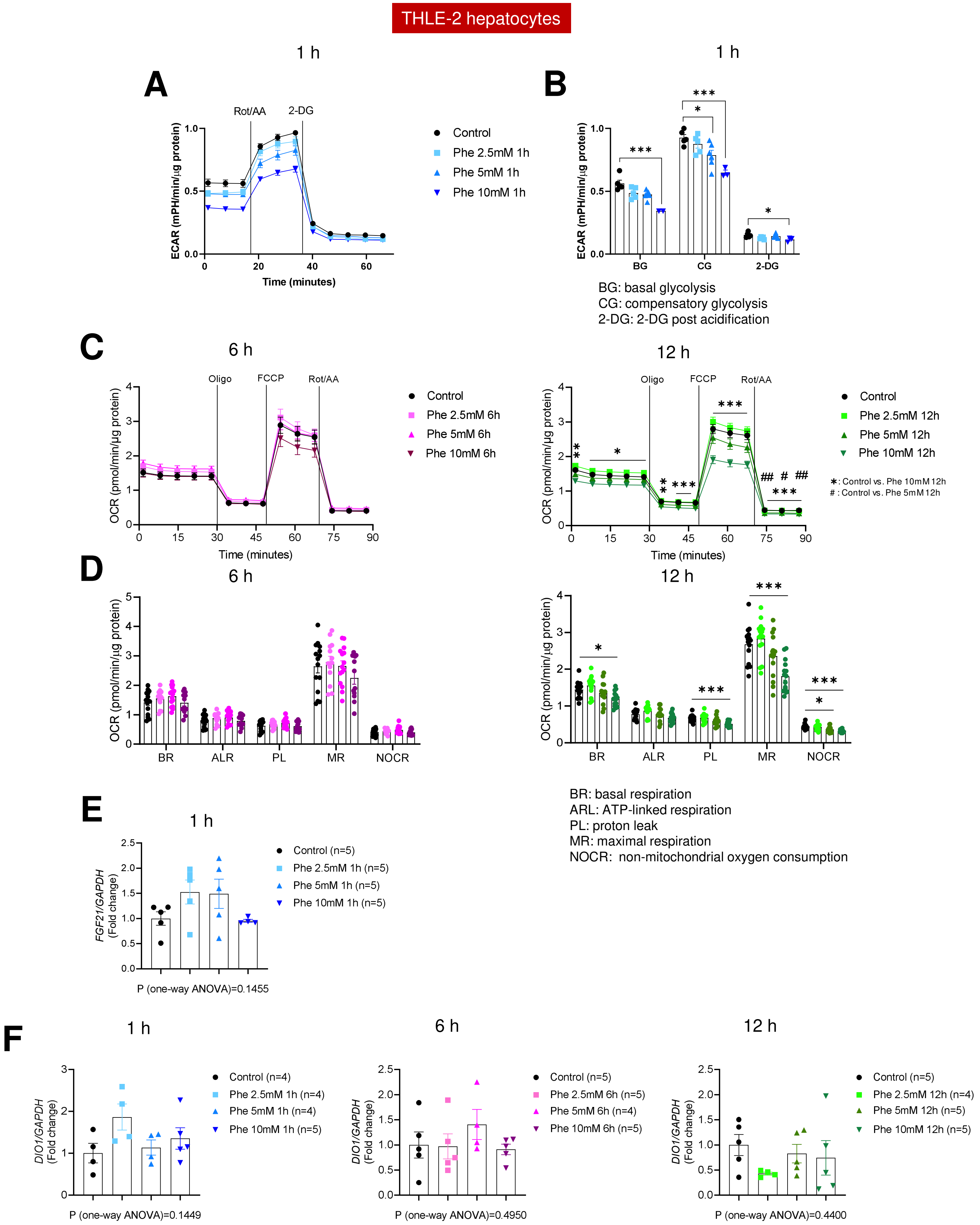
THLE-2 hepatocytes
A
1 h
Rot/AA
2-DG
ECAR (mPH/min/µg protein)
Time (minutes)
Control
Phe 2.5mM 1h
Phe 5mM 1h
Phe 10mM 1h
B
1 h
ECAR (mPH/min/µg protein)
BG
CG
2-DG
BG: basal glycolysis
CG: compensatory glycolysis
2-DG: 2-DG post acidification
C
6 h
Oligo
FCCP
Rot/AA
OCR (pmol/min/µg protein)
Time (minutes)
Control
Phe 2.5mM 6h
Phe 5mM 6h
Phe 10mM 6h
12 h
Control
Phe 2.5mM 12h
Phe 5mM 12h
Phe 10mM 12h
*: Control vs. Phe 10mM 12h
# : Control vs. Phe 5mM 12h
D
6 h
12 h
OCR (pmol/min/µg protein)
BR
ALR
PL
MR
NOCR
BR: basal respiration
ARL: ATP-linked respiration
PL: proton leak
MR: maximal respiration
NOCR: non-mitochondrial oxygen consumption
E
1 h
FGF21/GAPDH (Fold change)
Control (n=5)
Phe 2.5mM 1h (n=5)
Phe 5mM 1h (n=5)
Phe 10mM 1h (n=5)
P (one-way ANOVA)=0.1455
F
1 h
DIO1/GAPDH (Fold change)
Control (n=4)
Phe 2.5mM 1h (n=4)
Phe 5mM 1h (n=4)
Phe 10mM 1h (n=5)
P (one-way ANOVA)=0.1449
6 h
Control (n=5)
Phe 2.5mM 6h (n=5)
Phe 5mM 6h (n=4)
Phe 10mM 6h (n=5)
P (one-way ANOVA)=0.4950
12 h
Control (n=5)
Phe 2.5mM 12h (n=4)
Phe 5mM 12h (n=5)
Phe 10mM 12h (n=5)
P (one-way ANOVA)=0.4400

FIGURE S4. Effect of Phe on human hepatocytes

(A) Real time extracellular acidification rate (ECAR), **(B)** glycolytic parameters [basal (BG) and compensatory (CG) glycolysis and 2-deoxy-D-glucose (2-DG) post acidification], **(C)** oxygen consumption rate (OCR), **(D)** basal respiration (BR), ATP-linked respiration (ARL), proton leak (PL), maximal respiration (MR), non-mitochondrial oxygen consumption (NOCR), **(E)** fibroblast growth factor 21 *(FGF21)* mRNA levels and deiodinase 1 *(DIO1)* mRNA levels **(F)** in Transformed Human Liver Epithelial 2 (THLE-2) human hepatocytes after phenylalanine (Phe) treatments at different dosages (2.5, 5 and 10 mM) and times (1, 6 and 12 hours). Data are expressed as MEAN±SEM. Statistical significance was determined by One-way ANOVA followed of Bonferroni test or Kruskal-Wallis followed of Dunn's test; normality was assayed with Shapiro-Wilk test. Alpha level was set at 0.05. $^*P<0.05$, $^{***}P<0.001$. All statistical tests were two-sided.

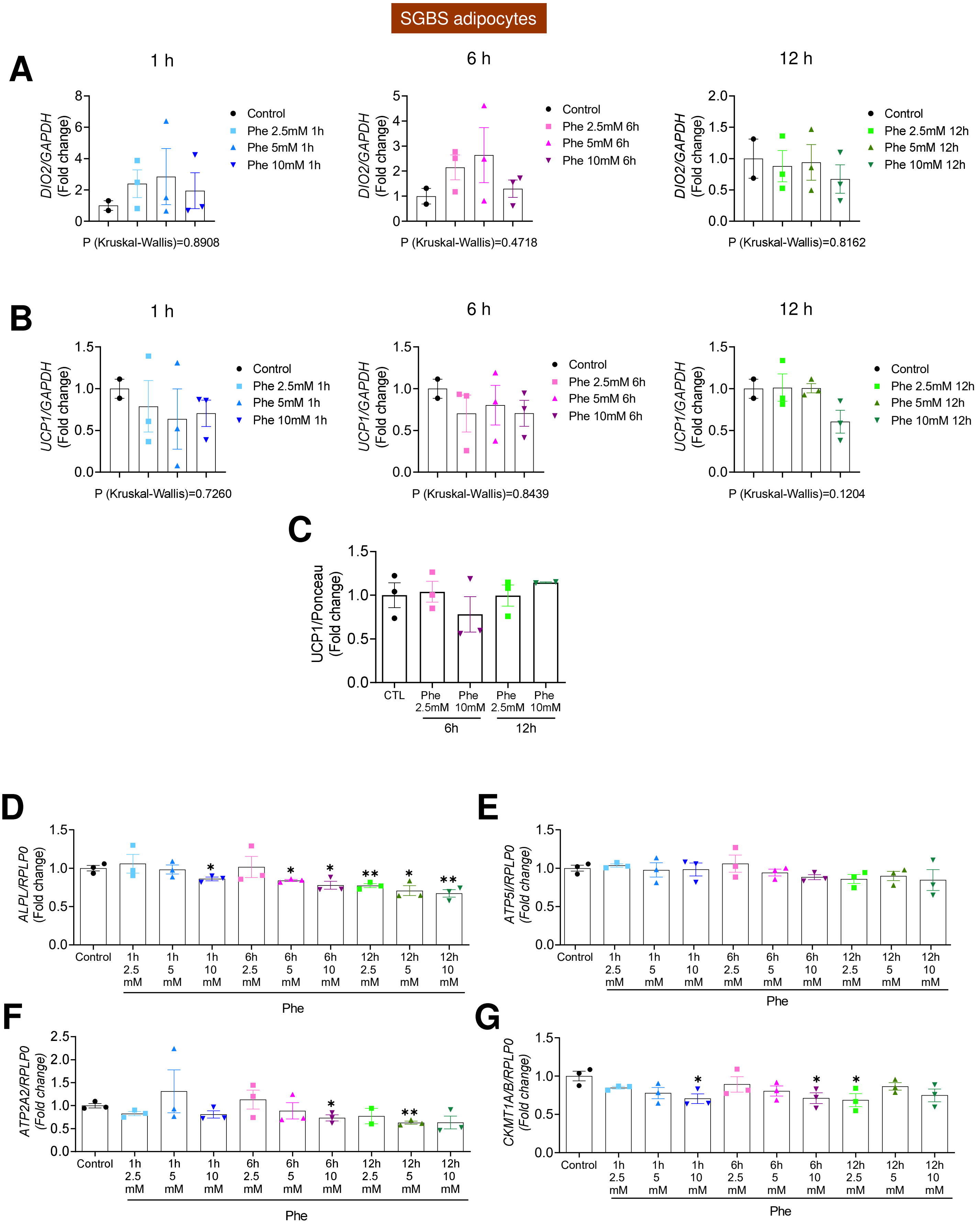
SGBS adipocytes
A
1 h
6 h
12 h
DIO2/GAPDH (Fold change)
Control
Phe 2.5mM 1h
Phe 5mM 1h
Phe 10mM 1h
Phe 2.5mM 6h
Phe 5mM 6h
Phe 10mM 6h
Phe 2.5mM 12h
Phe 5mM 12h
Phe 10mM 12h
P (Kruskal-Wallis)=0.8908
P (Kruskal-Wallis)=0.4718
P (Kruskal-Wallis)=0.8162
B
UCP1/GAPDH (Fold change)
P (Kruskal-Wallis)=0.7260
P (Kruskal-Wallis)=0.8439
P (Kruskal-Wallis)=0.1204
C
UCP1/Ponceau (Fold change)
CTL
Phe 2.5mM
Phe 10mM
6h
12h
D
ALPL/RPLP0 (Fold change)
E
ATP5I/RPLP0 (Fold change)
F
ATP2A2/RPLP0 (Fold change)
G
CKMT1A/B/RPLP0 (Fold change)
Control
1h 2.5 mM
1h 5 mM
1h 10 mM
6h 2.5 mM
6h 5 mM
6h 10 mM
12h 2.5 mM
12h 5 mM
12h 10 mM
Phe

FIGURE S5. Effect of Phe on human adipocytes

(A) Deiodinase 2 (DIO2; gene name *DIO2*) mRNA levels, **(B)** uncoupling protein 1 (UCP1; gene name *UCP1*) mRNA levels **(C)** UCP1 protein levels **(D)** tissue-nonspecific alkaline phosphatase (TNAP; gene name *ALPL*) mRNA levels, **(E)** ATP synthase subunit e (ATP5K; gene name *ATP5I*) mRNA levels **(F)** sarco/endoplasmic reticulum Ca2+-ATPase 2b (SERCA2b; gene name *ATP2A2*) mRNA levels and **(G)** creatine kinase, mitochondrial 1A/B (CKMT1; gene name *CKMT1A/B*) mRNA levels in Simpson-Golabi-Behmel Syndrome (SGBS) human adipocytes after phenylalanine (Phe) treatments at different dosages (2.5, 5 and 10 mM) and times (1, 6 and 12 hours). Glyceraldehyde-3-phosphate dehydrogenase (GAPDH; gene name *GAPDH*) was used as housekeeping gene to serve as an internal control. Data are expressed as MEAN±SEM. Statistical significance was determined by One-way ANOVA followed of Bonferroni test or Kruskal-Wallis followed of Dunn's test; normality was assayed with Shapiro-Wilk test. Alpha level was set at 0.05. *P<0.05, **P<0.01. All statistical tests were two-sided.

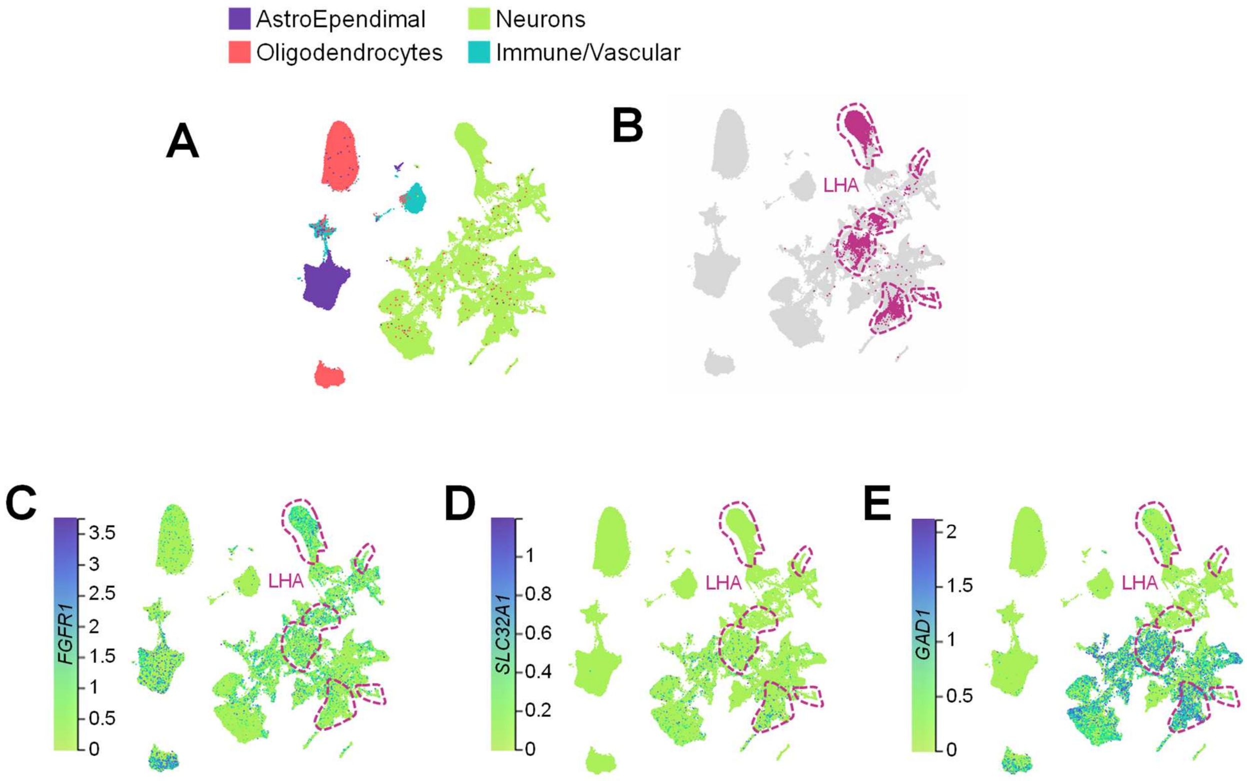

FIGURE S6. Expression of GABAergic and FGF21 receptor markers in the human lateral hypothalamic area

(A) Uniform manifold approximation and projection (UMAP) representation of major hypothalamic cell classes from the HypoMap single-cell RNA-sequencing dataset [41], **(B)** Localization of lateral hypothalamic area (LHA) neuronal clusters within HypoMap (magenta outline). **(C-E)** Expression of solute carrier family 32, GABA vesicular transporter, member 1 (SLC32A1; gene name *SLC32A1*; **C)**, glutamate decarboxylase 1 (GAD1; gene name *GAD1*; **D)** and FGF21 receptor (FGFR1; gene name *FGFR1*; **E)** across HypoMap, highlighting LHA neuronal populations.
